# Tonic pain modulates neural correlates of associative phasic pain memories

**DOI:** 10.1097/j.pain.0000000000003917

**Published:** 2026-01-21

**Authors:** Danielle Hewitt, Shuangyi Tong, Sarah Schreiber, Ben Seymour

**Affiliations:** a Wellcome Centre for Integrative Neuroimaging, University of Oxford, Oxford, United Kingdom; b Oxford Institute of Biomedical Engineering, University of Oxford, Oxford, United Kingdom

**Keywords:** Pain and nociception, Threat learning, Pavlovian conditioning, Pain anticipation, Electroencephalography, Event-related desynchronization/synchronization (ERD/ERS)

## Abstract

Tonic pain is proposed to maintain homeostatic integrity during recovery from injury. A key untested prediction of this homeostatic model is that it modulates internal representations of phasic pain. We investigated whether lateralised tonic pain modulates phasic pain-predictive cues on that side. Using a virtual-reality Pavlovian revaluation-style paradigm, we assessed physiological and neural conditioned responses with electroencephalography in theta, alpha, and beta frequency bands. Pain-predictive cues elicited neural enhanced alpha and beta suppression and increased pupil diameter during conditioning acquisition. Critically, tonic pain altered neural correlates of phasic conditioned responses during extinction, with reduced midfrontal theta synchronisation when the laterality of tonic pain was congruent with predicted phasic pain. These findings provide neural evidence for a topographically specific modulation of cue–pain representations by tonic pain, suggesting that tonic pain dynamically reshapes neural predictions of potential threat.

## Abstract 2

Supplemental Digital Content is Available in the Text.

Tonic pain modulates neural responses to pain-predictive stimuli, consistent with protective encoding of vulnerable body regions during sustained pain.

## 1. Introduction

Classical notions of pain propose that tonic and phasic pain subserve distinct functions in adaptive behaviour. Phasic pain enables rapid harm detection and nocifensive behaviours, which can be amplified by learning, allowing anticipation and avoidance through Pavlovian and instrumental conditioning.^62^ By contrast, tonic pain may enhance hyperprotective and recuperative behaviour during injury recovery, given increased vulnerability and changed homeostatic demands.^4,68^ This has led to the concept of tonic pain as a homeostatic state, relating to the widespread control of physiology and behaviour prioritising the maintenance of bodily integrity.

A cardinal, although untested, prediction of this homeostatic model is that tonic pain reshapes internal representations (memories) of phasic pain.^44^ This relationship is well-established in reward literature, where homeostatic states such as hunger or thirst reshape associative memories of food or drinks. In classical “devaluation” paradigms, animals learn to associate a cue with a particular food whilst hungry, but (Pavlovian) responding is diminished when sated.^11,30^ Such specificity is evidence of an adaptive, internal representation of the reward—“model-based” learning—as motivation is modulated by energy requirements.^14^ In the case of pain, lateralised tonic pain (eg, injury) should amplify previously learned associations with phasic pain on the affected side due to increased vulnerability to further damage. This requires introducing tonic pain during extinction to probe how homeostatic states modulate phasic pain memories, without confounding cue–pain associations. Selective modulation of neural predictive processing on the affected side ensures that these effects reflect topographically specific processing of the injured region, rather than nonspecific increases in arousal or attention.

Demonstrating the reshaping of neural representations of pain faces 2 methodological challenges. The first is imaging these representations. Electroencephalography (EEG) can reveal distinct neural signatures shaped by predictions and experiences. Aversive conditioned stimuli modulate EEG cortical oscillations in alpha (8-12 Hz) and theta (4-7 Hz) bands,^2,55,64^ and pain memories elicit suppressed alpha activity over parietal regions, implying somatomotor representations for pain-predictive cues.^1,46^ The second challenge is embedding associative memories into sufficiently threatening contexts. This can be solved using virtual reality (VR), where directional cues are experienced in three-dimensional space, integrating approaching cues with the spatial representation of tonic pain. Virtual reality thus provides ecological validity and precise spatial control essential for testing unilateral tonic pain effects on phasic pain associations.

We designed a paradigm to examine neural and physiological correlates of Pavlovian revaluation, using lateralised phasic pain conditioning and tonic pain, implemented in an EEG-VR study. During the task, we recorded EEG responses in theta (4-7 Hz), alpha (8-12 Hz), and beta (16-24 Hz) frequency bands alongside physiological arousal indicators (pupil diameter and gaze fixations). We predicted that pain cues would elicit heightened conditioned responses compared with neutral cues during acquisition, indexed by enhanced alpha and beta suppression and increased pupil diameter. Second, we predicted that lateralised tonic pain would modulate phasic conditioned responses to the same side during extinction. Given the exploratory nature of this analysis, we did not have a specific directional hypothesis.

## 2. Methods

### 2.1. Participants

Thirty-one pain-free participants (14 women) with no neurological conditions were recruited from the community and a pool of university staff and students. Five subjects were excluded during data collection: 3 due to technical issues during the recordings and 2 due to not adhering to the study protocol. The final sample included 26 participants (13 women; 24 right-handed, 1 ambidextrous) with a mean age of 26 ± 4.9 years (mean ± SD). The procedure was approved by the Research Ethics Committee of the University of Oxford, and all participants gave written informed consent at the start of the experiment following the Declaration of Helsinki. Participants were reimbursed with £60 on completion of the study.

### 2.2. Procedure

Experimental procedures were conducted in a single 3-hour session at the Oxford Institute of Biomedical Engineering. Participants were seated in a comfortable chair while EEG electrodes and the VR headset were applied. Participants were asked to hold the VR handsets with arms shoulder width apart, resting on an armrest raised at a 45° angle from the desk.

**Figure 1.**
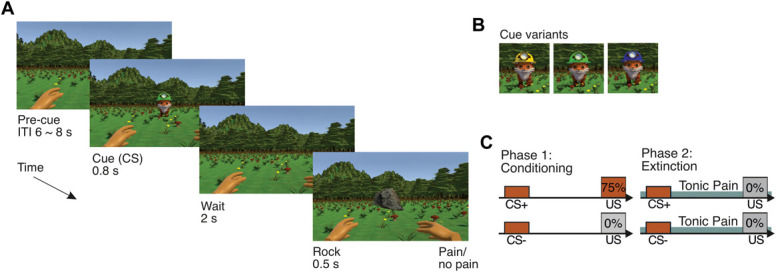
Pavlovian revaluation paradigm schematic. (A) At the trial onset, an animated fox wearing a green, yellow, or blue hat (conditioned stimulus [CS]; each colour corresponding to a cue condition) appeared centrally for 0.8 seconds, followed by a 2-second waiting period. Subsequently, a rock appeared and travelled with a left, right, or central trajectory toward the participant's hands (left/right) or the ground (middle), with a looming duration of 0.5 seconds, accompanied by a binaural sound increasing in volume as the object approached. Trials were followed by a random intertrial interval of 6 to 8 seconds. (B) Looming direction was determined by the fox's hat colour, with cues corresponding to each trial type counterbalanced for each participant. The unconditioned stimulus was the compound of the incoming rock and pain. (C) Pavlovian (trace) conditioning schematic. In phase 1 conditioning blocks, pain-related CS+ cues were followed by a 75% chance of experiencing phasic pain. Neutral CS− cues were followed by a 0% chance of experiencing pain. In phase 2 extinction blocks, no cues were followed by phasic pain. Tonic pain was delivered continuously throughout each extinction block using an upper arm pressure cuff to either the left or right arm. All participants received stimulation to both arms, with the side alternated across blocks in a counterbalanced order.

Participants viewed a realistic rendering of a simulated forest environment (see Fig. 1 for full details). The experiment consisted of 4 blocks, divided into 2 interspersed conditioning acquisition and extinction phases. During conditioning blocks, left and right looming objects were followed by electrical pain on the corresponding forearm with a 75% shock contingency (pain-related cues; determined automatically at the start of the experiment). No pain was delivered after central looming objects (neutral cues). A self-timed break followed every 24 trials, with 24 trials per cue (72 trials for each block; 144 trials overall).

During extinction blocks, tonic pain was applied continuously using a pressure cuff to either the left or right arm. Stimulation began immediately before the block began and was maintained until the self-timed intertrial break of least 1 minute for every 12 trials, when the pressure cuff was deflated. Each arm was stimulated in separate blocks, with the order counterbalanced across participants. During extinction trials, rocks travelled in the cued direction but disappeared 0.1 second early. No shocks were delivered. There were 108 trials for each block (216 trials overall, 36 for each cue type).

Throughout the task, trials were ordered pseudorandomly by shuffling the order of 6 trials (2 repetitions of each cue type) and repeating for the total trial number. During each break, participants were asked to rate the intensity and unpleasantness of electrical (conditioning blocks) or pressure (extinction blocks) pain stimuli on a scale of 0 (not painful/unpleasant), 3 (just painful/unpleasant), to 10 (maximum pain/unpleasantness) for each site. At the end of the experiment, participants were asked to complete a brief questionnaire regarding demographic characteristics and the State-Trait Anxiety Inventory.

### 2.3. Pain stimuli

#### 2.3.1. Phasic electrical pain stimuli

Electrical pain stimulation was applied to the skin of the forearm using EPS-P10 electrodes (MRC Systems GmbH, Heidelberg, Germany) connected to a constant current stimulator (Digitimer DS7a, Digitimer Ltd, Welwyn Garden City, United Kingdom). Electrical stimulation intensity was set for each participant as a multiple of electrical detection threshold (EDT). Electrical detection threshold was determined for each forearm at the start of the experiment using the method of limits, where single electrical stimuli were delivered in descending and ascending steps of 0.002 mA to establish the lowest perceptible threshold. Participants rated single electrical stimuli at a multiple of EDT (10-30×) until an intensity of 5/10 on a scale of not painful (0), just painful (3), to most pain imaginable (10) was reached, following previous studies.^5,33,41^ During the main experiment, stimuli consisted of 2 rapidly succeeding pulses (frequency 1 Hz, pulse width 1 ms, duration 1 ms) at the test stimulation intensity. If participants reported that the stimuli were no longer painful during breaks, intensity was increased; similarly, if the stimuli became intolerable, intensity was decreased.

#### 2.3.2. Tonic pressure pain stimuli

Tonic pain was delivered to the left and right upper arms using a manual pneumatic blood pressure cuff during extinction blocks only. Each extinction block involved stimulation of only one arm, with stimulation side alternating between blocks in a counterbalanced order across participants. Pressure was initiated immediately before the block began and maintained throughout the block until the scheduled self-timed break, at which point the cuff was deflated. Nociceptive responses elicited by pressure stimuli have been demonstrated to originate from deep tissue muscle strain rather than superficial cutaneous or ischemic sensations.^21,25^ Stimulation intensity was determined at the start of the experiment using a staircase procedure, where the pressure was slowly increased until participants reported a “just painful” sensation (3/10).

### 2.4. Virtual-reality and electroencephalography equipment

The experiment was designed in Unity and viewed with a HTV Vive Pro Eye headset, connected to SteamVR using an Alienware PC. Eye-tracking data were collected using the headset following calibration at the start of the experiment.

#### 2.4.1. Physiological data analysis

Physiological VR data were processed in Jupyter using Python 3. Trial data were extracted from continuous files for each participant for the whole trial period, starting −1 second before cue presentation to 3.3 seconds after cue onset. Data were interpolated to account for slight variations in sampling rate throughout the trials, to a total of 3300 data points per trial.

Pupil diameter for the left and right pupils was extracted from trial data. To account for ocular artefacts such as blinks, data points exceeding 2 SDs from the trial mean were identified. Where 150 consecutive extreme data points were found (0.15 seconds), data points were removed and linearly interpolated following previous studies^45,48,61^ and according to minimum blink duration.^65^ Trials containing ≥25% interpolated data were removed from further analysis (0 trials). Cleaned data were individually baseline corrected (−250 to −750 ms) by subtracting the average of the baseline period from the trial period,^45,61^ and data from the right and left eyes were subsequently averaged. After visual inspection, the anticipation interval was divided into two 1 second segments for analyses: early (800-1799 ms) and late (1800-2799 ms) anticipation, in line with literature on the temporal dynamics of conditioning and anticipation effects.^1,2^

The number of fixations on the looming rock were extracted from the last 500 milliseconds of the trial and averaged for each condition. Gaze direction and hand position were extracted from trial data. No significant changes in gaze direction or mean displacement of hand position were observed throughout (full methods and results in Supplementary Materials, http://links.lww.com/PAIN/C449) (*P* > 0.05).

#### 2.4.2. Electroencephalography acquisition

Whole-scalp EEG was continuously recorded using a 32-channel system (BrainProducts GmbH, Munich, Germany). Actively shielding Ag-AgCl electrodes were mounted on an electrode cap (actiCap snap, BrainProducts) according to the International 10–20 system.^32^ The cap was aligned with 3 anatomical landmarks of 2 preauricular points and the nasion. Electrolyte gel was applied to achieve electrode-to-skin impedances below 25 kΩ throughout the experiment. A recording band-pass filter was set at 0.001 to 131 Hz. The sampling rate was 500 Hz, corresponding to a sampling interval of 2000 µs. Electrode FCz was used as a reference electrode, and electrode FPz was used as the ground electrode. Electroencephalography average reference was applied, and signals were digitised with a LiveAmp amplifier, connected to BrainVision Recorder 1.25 running on an Alienware PC. Owing to a technical issue with the Bluetooth connection between the LiveAmp and the PC, 0.64 ± 1.86 trials were lost for each participant and block.

#### 2.4.3. Spectral analysis of electroencephalography signals

EEG data were processed using EEGLab.^15^ Continuous EEG data were split into 8-second epochs (−2.5 to 5.5 seconds around cue onset). Data were re-referenced to the common average^40^ and filtered from 1 to 70 Hz, with a notch filter from 48 to 52 Hz. Data for all 4 conditions were subsequently merged, resulting in one datafile per participant.

Oculomotor and muscular artefacts were removed from the data using independent component analysis (ICA). For optimal ICA,^43^ a subset of the data filtered from 1 to 30 Hz was created for each participant. RUNICA was conducted on the streamlined datafile, and ICA weights were exported and backprojected onto the original merged datafile. Data were epoched from −2.5 to 3.5 seconds around cue onset. Electrode channels with large artefacts were identified with visual inspection and interpolated to a maximum of 10% of all electrodes. Epochs containing extreme data due to movement or muscle artefacts were excluded using a semiautomated method. Outlier values were identified using *pop_eegthresh*.*m* with limits of −125 to 125 µV during the entire epoch. Improbable data were marked using *pop_jointprob*.*m* using single channel and global channel limits of 7 SDs. Data were visually inspected and marked trials manually reviewed. Average epochs remaining after artefact correction for each block were: conditioning 69.56 ± 3.55, extinction 104.06 ± 3.68. Accepted trials were not significantly different between cue types (F [2,309] = 0.013, *P* = 0.987).

To enhance small local currents and arrive at reference-free data, data were transformed into current source density using a Laplacian Spherical Spline interpolation method^29,50^ implemented in FieldTrip^49^ (http://fieldtriptoolbox.org) using default regularisation (lambda 1e-5) and skin conductivity (0.33 S/m) parameters and 9 degrees of Legendre polynomials, as recommended by FieldTrip for data sets with ≤32 channels. Power spectral densities were computed using a discrete Fourier time-frequency transformation using the Welch method in 1 second windows shifted in overlapping 0.1 second segments to yield a power time series of 61 points (1-70 Hz, frequency resolution 1 Hz). Data were smoothed using a 4-Hz multitaper. Relative power was evaluated using the classical event-related desynchronization (ERD) transformation,

$$D\%=\left(100\times \frac{A-R}{R}\right)$$

where *D* represents the percentage power change during epochs after cue onset (*A*) relative to the reference period (*R*, −2 to −1 seconds).^53^ Positive and negative *D* values correspond to power increases (event-related synchronization [ERS])^51^ and decreases (ERD),^52,54^ respectively.

Relative band power in alpha (8-12 Hz), beta (16-24 Hz), and theta (4-7 Hz) frequency bands was extracted from all 32 active scalp electrodes in 3 timebins: 0 to 0.79 seconds, 0.8 to 1.79 seconds, and 1.8 to 2.8 seconds (cue, early, and late anticipation, respectively). Electrodes were clustered into 4 spatially adjacent regions: frontal (Fp1, Fp2, Fz), central (FC5, FC1, FC2, FC6, C3, Cz, C4), parietal (CP5, CP1, CP2, CP6, P7, P3, Pz, P4, P8), and occipital (O1, Oz, O2), based on previous literature showing prominent band power changes during spatial attention, aversive learning, and pain anticipation.^1,2,19,27^

### 2.5. Statistical analysis

#### 2.5.1. Self-reported measures

For each tonic and phasic pain, two 2 × 2 × 3 ANOVAs (left vs right side; within-block rating time; block repetition) (conditioning/extinction block 1 or 2) assessed changes in pain intensity and pain unpleasantness using SPSS v. 29 (IBM Corp., Amonk, NY). For significant main effects, Bonferroni-corrected pairwise comparisons are reported. Repeated-measures ANOVAs and corresponding pairwise comparisons are reported for interactions.

#### 2.5.2. Electroencephalography and physiological data

Event-related desynchronization/event-related synchronization and physiological data were entered into individual LMMs in RStudio (version 2023.12.1.402).^58^ LMMs were computed with *lme4*^3^ and *lmerTest* packages^37^ using Satterthwaite approximation for degrees of freedom. Before LMMs, data were checked for extreme values to minimise the influence of outliers on model estimates. Extreme values were identified using z-scores, with values beyond ±3 standard deviations from the mean removed.

Core analyses central to the study hypotheses were conducted: (1) to investigate behavioural and EEG evidence of conditioning, and (2) to examine the effects of tonic pain-conditioned stimulus [CS+] congruency during extinction. Therefore, separate LMMs were assessed: (1) changes as a function of block (conditioning and extinction) and cue (neutral, pain-related), and (2) changes as a function of tonic pain side. Both LMMs additionally included measurement interval (cue, early, late, and approach timebins). The approach timebin was not included in EEG analyses due to the presence of moving stimuli. For EEG data, LMMs were constructed individually for alpha (8-12 Hz), beta (16-24 Hz), and theta (4-7 Hz) frequency bands and electrode clusters, as it was expected that relative band power would differ between frequency bands and topographically over the scalp.

Electroencephalography lateralisation effects were tested for all electrode clusters by including hemisphere as a fixed effect to assess cortical activation changes in left- and right-hemisphere electrodes as a function of cue direction and tonic pain side. These analyses did not reveal significant hemisphere effects or interactions for any cluster (see Supplementary Materials, http://links.lww.com/PAIN/C449). To simplify presentation and reduce the number of multiple comparisons, left and right hemisphere electrodes within each cluster were therefore pooled for the main analyses.

For all LMMs, participants and block repetition were modelled as hierarchical random effects. Models were estimated using ML and nloptwrap optimizer. Fixed effects structures were determined stepwise by including interaction terms or predictors in the model. Predictors and interaction terms which did not significantly change the variance explained were removed to avoid overparameterisation. Bayesian Information Criterion and Akaike Information Criterion were compared for each model to find the simplest model that explained the data.

Exploratory analyses investigated the influence of covariates on the effect of tonic pain–cue congruency. Covariates of interest were age, sex, STAI trait score, mean intensity, and mean unpleasantness of tonic pain. Each predictor was added to a covariate-only model. Covariates that significantly predicted change in the measured variable were included in LMMs with the factor of tonic pain side. For clarity of presentation, these results are described in Supplementary Materials, http://links.lww.com/PAIN/C449.

*P*-values for all main effects of interest and interactions were adjusted using family-wise error (FWE) correction. Significant main effects which surpassed FWE-correction were followed up with pairwise comparisons using the *emmeans* package. Significant interactions were followed up with interaction contrasts for estimated means. All contrasts were adjusted for multiple comparisons using the Šidák correction.

#### 2.5.3. Computational model analysis of trial-by-trial anticipatory learning

Computational learning models were used to capture pain learning in ERD/S and pupil dilation at the trial level. We constructed a standard temporal difference reinforcement learning model, as previously implemented for human pain conditioning.^72,73^ The model is a simple, “real-time” instantiation of the Rescorla-Wagner model,^60^ where the value *V* of trial *n* + 1 for a cue *j* is updated based on the value of current trial *n* and the prediction error, the difference between current value *V*_*j*_, and outcome stimulus value *R* at trial *n*, weighted by a constant learning rate α:

$$V_{j}\left(n+1\right)=V_{j}\left(n\right)+\alpha \left(R\left(n\right)-V_{j}\left(n\right)\right)$$

where the learning rate α (0 ≤ α ≤ 1) is a free parameter. R(n) was coded as +1 for pain outcomes and 0 for no pain outcomes. Thus, higher value estimates (*V*) reflect greater expected pain associated with a given cue, based on prior outcomes. Regression analyses (ordinary least-squares) were implemented separately for EEG (on a compound variable of frequency, time, and cluster) and physiological data to assess the relationship between learned model values and measured responses during conditioning and extinction blocks, and during extinction blocks based on tonic pain laterality. *P*-values for all effects were adjusted using FWE-correction.

## 3. Results

Twenty-six human volunteers participated fully in the task. In the first stage, participants underwent Pavlovian trace conditioning to left- or right-sided painful shocks. In the second stage, tonic pain was applied to one side using an upper arm pressure cuff. The cues were then shown in extinction (such that the subject never simultaneously experienced phasic and tonic pain—a core requirement for demonstrating de-/revaluation).

In basic analyses, there were no effects of laterality on pain ratings. Phasic electrical and tonic pressure stimuli were rated as painful (phasic 4.45 ± 1.28, tonic 3.61 ± 1.09) and unpleasant (phasic 4.62 ± 1.36, tonic 4.35 ± 1.31) throughout the experiment. As expected, there were no significant effects of laterality (left vs right) on tonic or phasic pain ratings (*P >* 0.05). The effect of time (within- and between-blocks) on pain ratings and unpleasantness was significant for both tonic and phasic pain, with reduced ratings over time and between blocks (*P <* 0.05; Supplementary Materials, http://links.lww.com/PAIN/C449).

### 3.1. Behavioural evidence of conditioning

Physiological measures provided evidence that participants learned to associate cues (CS+) with pain (US). Pupil diameter and gaze fixations were entered into individual LMMs to assess changes as a function of block (conditioning and extinction), cue (neutral, pain-related), and measurement interval (cue, early anticipation, late anticipation, and rock approach timebins). Full results are summarized in Table 1.

**Table 1:** Fixed effects for physiological comparisons of interest.

Measure	Predictor	*b*	*SE*	*df*	*t* value	*P*
Pupil diameter (pupil diameter ∼ cue × block × timebin)	Intercept	−0.04	0.01	367.22	−3.65	0.000
	Cue	−0.03	0.01	1171.86	−2.58	0.010*
	Block	0	0.01	1171.77	−0.21	0.834
	Timebin	0	0	1171.9	0.77	0.441
	Cue × condition	−0.01	0.01	1171.75	−0.5	0.618
	Cue × timebin	0.02	0	1171.92	3.68	0.000*
	Block × timebin	0	0	1171.9	0.06	0.95
	Cue × block × timebin	0.01	0	1171.87	1.89	0.059
Pupil diameter (pupil diameter ∼ TonicSide + timebin)	Intercept	−0.08	0.01	138.66	−7.90	0.000
	Congruency	0.01	0.01	764.67	1.21	0.226
	Timebin	0.02	0	763.96	7.27	0.000*
Fixations (fixations ∼ cue × block)	Intercept	0.07	0.02	45.38	4.13	0.000
	Cue	0.12	0.01	256.47	10.43	0.000*
	Block	0.00	0.01	255.33	−0.14	0.890
	Cue × block	−0.02	0.02	255.38	−1.20	0.230
Fixations (fixations ∼ TonicPain)	Intercept	0.19	0.02	27.16	8.06	0.000
	Congruency	−0.01	0.01	154.53	−0.96	0.338

* Effects that survived FWE correction (*P* < 0.05). Sum contrast coding was used for block. Default (treatment) contrast coding was used for cue (reference: neutral), measurement timebin (reference: cue) and tonic pain side (reference: incongruent).

FWE, family-wise error.

**Figure 2.**
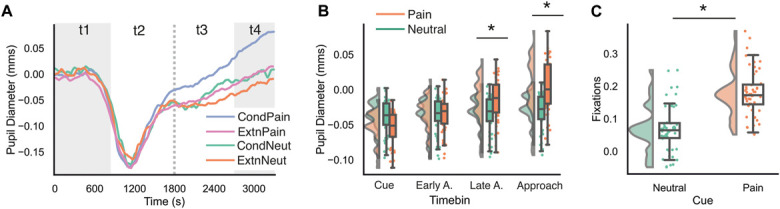
Physiological evidence of conditioning. (A) Grand-average changes in pupil diameter compared with the pretrial baseline are plotted continuously over the entire trial, with shaded areas representing CS presentation (t1) and rock approach (t4). The dotted line represents separation of the CS-US interval into early (t2) and late anticipation (t3) timebins. (B) Mean pupil diameter was averaged within each of the 4 discrete timebins for analysis. (C) Fixations on the approaching rock during the approach period (t4) were averaged and compared for neutral and pain-related cues. Box-plots show interquartile ranges (centre line at the median, upper and lower bounds at 75th and 25th percentiles), with whiskers at minimum and maximum values within 1.5 × interquartile range. Asterisks indicate significant results at *P* < 0.05 following family-wise error correction. CS, conditioned stimulus.

For pupil diameter (Fig. 2A), LMMs showed a significant main effect of cue, due to more dilated pupils for pain vs neutral cues (t [1180] = −1.90, *P* = 0.058, n.s. in corrected pairwise comparisons). This result was qualified by a cue × timebin interaction. Pairwise contrasts showed that pain cues elicited significantly greater pupil dilation compared with neutral cues in late anticipation (t [1180] = −3.23, *P* = 0.001) and approach timebins (t [1180] = −4.07, *P* < 0.001; Fig. 2B), but not during cue presentation or early anticipation (*P >* 0.05). The main effect of block was not significant (*P >* 0.05).

For fixations, participants directed significantly more fixations toward looming rocks after pain-related compared with neutral cues (t [260] = −13.43, *P* < 0.001; Fig. 2C). No significant effects of block or cue × block interactions were observed.

### 3.2. Neural conditioned responses

**Figure 3.**
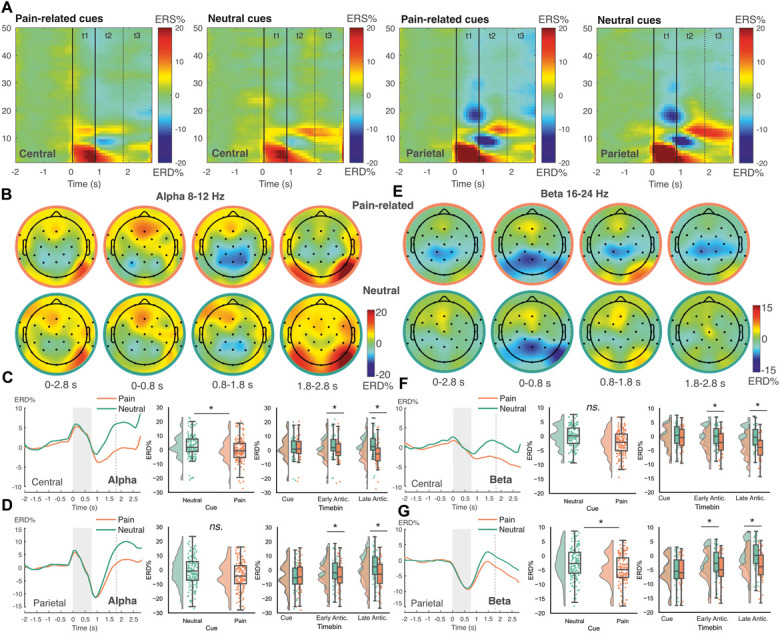
Neural evidence of conditioning I: the effect of cue on EEG band power. (A) Time–frequency maps depicting relative band power changes in central and parietal regions after pain-related (CS+) vs neutral cues. Topographic maps display ERD/S (event-related desynchronization/synchronization) in alpha and beta frequency bands averaged over the whole trial before US onset (0-2.8 seconds), as well as during cue presentation (0-0.79 seconds), early anticipation (0.8-1.79 seconds), and late anticipation (1.8-2.8 seconds) intervals. (B and C) Alpha band power in central electrodes was significantly modulated by cue, with stronger ERD after pain-related (CS+) compared with neutral cues. Significant cue × timebin interactions were observed in the alpha band in central (C) and parietal (D) regions, with cue effects at early and late anticipation intervals. (E-G) Beta band power in parietal sites was also significantly modulated by cue, with stronger ERD after pain-related compared with neutral cues. Cue × timebin interactions in the beta band in central (F) and parietal (G) regions indicated that cue effects emerged during early and late anticipation intervals. Time courses illustrate band power changes for each cue type, averaged over blocks. Shaded regions and dotted lines illustrate cue presentation and separation into early and late anticipation intervals, respectively. Box plots indicate the interquartile range (median = centre line; boxes = 25th-75th percentile; whiskers = values within 1.5 × interquartile range). Negative values indicate ERD; positive values indicate ERS. Asterisks denote significant effects at *P* < 0.05 (family-wise error corrected). CS, conditioned stimulus; EEG, electroencephalography; ERD, event-related desynchronization; ERS, event-related synchronization.

EEG results supported evidence of conditioning (Fig. 3A). LMMs were fit for each frequency band and electrode cluster to assess changes as a function of block (conditioning, extinction), cue (neutral CS−, pain-related CS+), and measurement interval (cue, early anticipation, and late anticipation). Full results are summarized in Table 2.

**Table 2:** Fixed effects for electroencephalography comparisons of interest.

Cluster	Predictor	Band														
		Alpha					Beta					Theta				
		*b*	*SE*	*df*	*t* value	*P*	*b*	*SE*	*df*	t value	*P*	*b*	*SE*	*df*	*t* value	*P*
Frontal	Intercept	9.79	2.63	215.08	3.72	0	2.91	1.56	270.21	1.87	0.063	27.11	2.8	442.17	9.69	0
	Cue	2.78	2.65	2696.52	1.05	0.293	−1.1	1.61	2756	−0.68	0.496	−2.34	3.05	2646.57	−0.76	0.444
	Block	−6.16	3.05	2696.48	−2.02	0.044	−1.58	1.86	2756	−0.85	0.398	−11	3.51	2646.3	−3.14	**0.002** *
	Timebin	−2.12	1	2696.51	−2.12	0.034	−0.08	0.61	2756	−0.13	0.899	−8.68	1.15	2646.07	−7.57	**0.000** *
	Cue × block	2.74	3.73	2696.46	0.73	0.463	3.02	2.28	2756	1.32	0.186	4.3	4.31	2646.56	1	0.318
	Cue × timebin	−2.65	1.22	2696.56	−2.17	0.03	−0.71	0.75	2756	−0.95	0.343	−0.31	1.41	2646.09	−0.22	0.828
	Block × timebin	1.68	1.41	2696.64	1.19	0.234	0.05	0.86	2756	0.06	0.951	3.24	1.62	2646.23	2	0.045
	Cue × block × timebin	0.84	1.73	2696.62	0.49	0.625	−0.55	1.06	2756	−0.52	0.601	−0.76	1.99	2646.38	−0.38	0.703
Central	Intercept	1.79	2.16	86.55	0.83	0.408	−0.17	1.3	210.01	−0.13	0.898	18.87	1.65	307.44	11.41	0
	Cue	4.97	1.8	6410.88	2.76	**0.006** *	2.67	1.3	6490.02	2.06	0.039	1.45	1.73	6393.64	0.84	0.404
	Block	−2.24	2.08	6410.85	−1.08	0.282	1.52	1.5	6490.02	1.02	0.309	−6.39	2	6393.99	−3.19	**0.001** *
	Timebin	0.34	0.68	6410.92	0.5	0.614	−0.01	0.49	6490.01	−0.02	0.982	−6.25	0.65	6393.58	−9.57	**0.000** *
	Cue × block	−3.76	2.55	6410.9	−1.48	0.14	−3.97	1.83	6490.02	−2.16	0.03	0.09	2.45	6393.84	0.04	0.971
	Cue × timebin	−4.2	0.84	6411.23	−5.02	**0.000** *	−2.71	0.6	6490.01	−4.52	**0.000** *	−0.77	0.8	6393.82	−0.96	0.337
	Block × timebin	1.22	0.96	6411.01	1.26	0.207	−0.65	0.69	6490.02	−0.94	0.349	2.18	0.93	6393.95	2.35	**0.019** *
	Cue × block × timebin	2.34	1.18	6411.18	1.98	0.048	2.47	0.85	6490.02	2.91	**0.004** *	0.11	1.13	6393.92	0.1	0.923
Parietal	Intercept	−6.27	2.48	71.14	−2.53	0.014	−8.56	1.34	89.81	−6.37	0	20.78	1.82	147.48	11.45	0
	Cue	4.38	1.94	8042.53	2.26	0.024	3.24	1.13	8364.99	2.86	**0.004** *	1.19	1.71	8136.99	0.69	0.489
	Block	−3.13	2.23	8041.92	−1.4	0.161	1.1	1.31	8364.99	0.84	0.398	−5.25	1.98	8137.42	−2.66	**0.008** *
	Timebin	2.37	0.73	8041.9	3.23	**0.001** *	2.61	0.43	8364.99	6.09	**0.000** *	−7.52	0.64	8137.03	−11.69	**0.000** *
	Cue × block	−3.09	2.73	8042.17	−1.13	0.258	−3.51	1.6	8364.99	−2.19	0.029	−1.18	2.42	8137.2	−0.49	0.625
	Cue × timebin	−3.54	0.9	8042.61	−3.94	**0.000** *	−2.52	0.52	8364.99	−4.82	**0.000** *	−0.75	0.79	8137	−0.95	0.343
	Block × timebin	2.24	1.03	8041.97	2.17	0.03	0.19	0.61	8364.99	0.31	0.754	2.9	0.91	8137.17	3.19	**0.001** *
	Cue × block × timebin	1.42	1.27	8042.25	1.12	0.264	1.53	0.74	8364.99	2.07	0.039	0.06	1.11	8137.2	0.06	0.956
Occipital	Intercept	−3.24	2.99	196.13	−1.09	0.279	−4.23	1.69	192.31	−2.5	0.013	18.91	2.5	213.08	7.56	0
	Cue	2.77	2.98	2661.63	0.93	0.352	−0.44	1.67	2745	−0.27	0.791	0.06	2.52	2723.69	0.02	0.981
	Block	0.03	3.42	2661.2	0.01	0.992	−2.26	1.93	2744.99	−1.17	0.243	−2.31	2.91	2724.13	−0.79	0.427
	Timebin	1.37	1.12	2661.08	1.22	0.221	1.62	0.63	2744.99	2.56	**0.011** *	−8.01	0.95	2724.09	−8.47	**0.000** *
	Cue × block	−5.04	4.19	2661.26	−1.2	0.23	1.02	2.37	2744.99	0.43	0.667	−0.72	3.56	2723.97	−0.2	0.84
	Cue × timebin	−2.2	1.38	2661.46	−1.59	0.111	0.29	0.77	2745.02	0.38	0.705	−0.32	1.16	2723.68	−0.28	0.781
	Block × timebin	1.53	1.59	2661.53	0.96	0.337	1.02	0.89	2744.99	1.14	0.253	2.56	1.34	2723.97	1.91	0.056
	Cue × block × timebin	1.48	1.95	2661.42	0.76	0.449	−0.91	1.1	2745.01	−0.83	0.408	−0.58	1.64	2723.91	−0.35	0.724

* Effects that survived FWE correction (*P* < 0.05). Default (treatment) contrast coding was used for variables of block (reference: conditioning), cue (reference: neutral), and measurement timebin (reference: cue).

FWE, family-wise error.

In the *alpha band*, pain-related cues elicited stronger ERD than neutral cues at central electrodes (Figs. 3B and C) (t [6418] = 6.15, *P <* 0.001). This effect was modified by cue × timebin interactions over central and parietal regions (Figs. 3C and D). Pairwise contrasts showed stronger central and parietal ERD to pain-related cues during early anticipation (central, t [6418] = 6.17, *P <* 0.001; parietal, t [8050] = 5.5, *P <* 0.001) and late anticipation timebins (central, t [6418] = 7.87, *P <* 0.001; parietal, t [8050] = 6.92, *P <* 0.001). Comparatively, no cue effect was present during cue presentation (*P >* 0.05).

In the *beta band*, pain-related cues also evoked stronger ERD compared with neutral cues, particularly in parietal regions (Fig. 3E; t [8372] = 6.71, *P <* 0.001). This effect was qualified by a cue × timebin interaction in central and parietal regions. Similar to the alpha band, anticipatory intervals drove the difference, with sustained ERD for pain-related trials across both early and late anticipation. In central sites (Fig. 3F), ERD developed throughout the trial for pain cues only, with significant differences based on cue valence appearing during early and late anticipation (early, t [6497] = 6.54, *P <* 0.001; late, t [6497] = 6.83, *P <* 0.001). In parietal sites (Fig. 3G), beta ERD during cue presentation was sustained for pain cues only (ie, attenuated for neutral cues) throughout early and late anticipation intervals (cue, *P >* 0.05; early, t [8372] = 6.71, *P <* 0.001; late, t [8372] = 7.92, *P <* 0.001).

**Figure 4.**
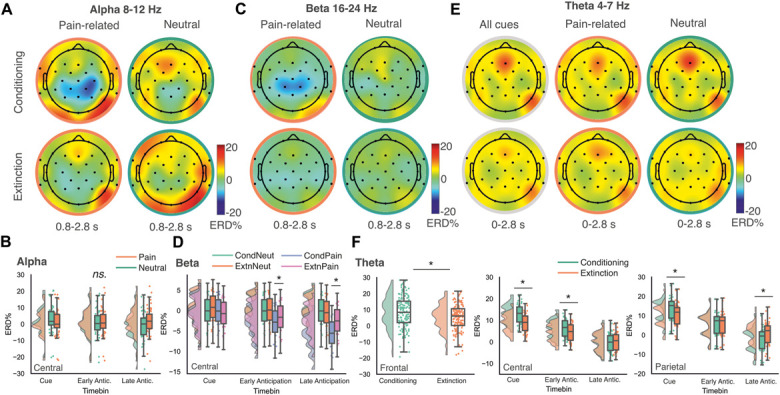
Neural evidence of conditioning II: the effect of conditioning block on EEG band power. Topographic maps show ERD/S (event-related desynchronization/synchronization) in response to pain-related vs neutral cues in phase 1 conditioning (top row) and phase 2 extinction blocks (bottom row). Alpha and beta maps are plotted during early and late anticipation intervals (0.8-2.8 seconds), while theta maps are plotted throughout the anticipatory period (0-2.8 seconds), due to significant results in the respective timebins. (A and B) Alpha band power was not modulated by block, nor was there a significant block × cue interaction. (C and D) Beta band power. (C) Topographic maps show stronger ERD for pain-related cues during conditioning compared with extinction over central sites. (D) Central beta ERD exhibited a significant cue × timebin × block interaction, with stronger ERD for pain cues during early and late anticipation in the conditioning block. (E and F) Theta band activity. (E) Topographic maps illustrate stronger ERS across frontal and central areas during conditioning vs extinction. (F) Box plots show significant main effects of block and block × timebin interactions in central and parietal regions, with stronger ERS during cue presentation and early anticipation in conditioning blocks that diminished over time. Box plots indicate the interquartile range (median = centre line; boxes = 25th–75th percentile; whiskers = values within 1.5 × interquartile range). Negative values indicate ERD; positive values indicate ERS. Asterisks denote significant effects at *P* < 0.05 (family-wise error corrected). EEG, electroencephalography; ERD, event-related desynchronization; ERS, event-related synchronization.

The effect of block (conditioning vs extinction) was investigated in alpha, beta, and theta frequency bands. While in the alpha band, the effect of block and cue × block interactions were not significant (all *P >* 0.05; Figs. 4A and B), beta band power was modulated by block. Although the main effect of block was not significant (*P >* 0.05), a significant cue × timebin × block interaction was observed over central sites (Fig. 4C). Pairwise comparisons showed that differences in the processing of pain-related cues between conditioning and extinction were significant during early and late anticipation, with greater ERD for pain-related cues in conditioning blocks (early, t [6497] = −3.00, *P =* 0.003; late, t [6497] = −4.78, *P <* 0.001; Fig. 4D). No difference between conditioning and extinction blocks was found during cue presentation or for neutral cues (*P >* 0.05).

In the *theta band*, cue effects were not significant (*P >* 0.05). However, a main effect of block was observed, with stronger ERS during conditioning vs extinction over frontal and central electrodes (frontal, t [2655] = 3.83, *P* < 0.001; central, t [6401] = 4.06, *P <* 0.001; Figs. 4E and F). A significant main effect of block was also observed in parietal regions, although this effect was not significant in corrected pairwise comparisons (*P >* 0.05). Block effects were modulated by timebin, with significant interactions in central and parietal regions. In parietal regions, this effect was due to strong ERS during cue presentation which was greater in conditioning vs extinction blocks, and which reduced throughout the trial, manifesting as stronger ERD during late anticipation (cue, t [8145] = 4.03, *P* < 0.001; late, t [8145] = −4.14, *P <* 0.001). In central regions, this effect was due to stronger ERS during cue presentation and early anticipation for conditioning vs extinction blocks (cue, t [6401] = 5.61, *P* < 0.001; early, t [6401] = 4.07, *P <* 0.001). No block differences were observed during late anticipation (*P >* 0.05). The cue × block interaction was not significant (*P >* 0.05).

### 3.3. Computational modelling of trial-by-trial anticipatory learning signals

We examined whether neurophysiological signals tracked cue–outcome predictions across conditioning and extinction blocks on a trial-by-trial basis. Full results are presented in Supplementary Materials, http://links.lww.com/PAIN/C449. Although overall model fits explained a relatively small proportion of variance (adj. R^2^ ≈ 0.02-0.03), regression analyses revealed statistically significant relationships between predicted values and both pupil dilation and EEG oscillations.

For pupil diameter, the model significantly predicted trial-by-trial changes (F [7,35239] = 155.7, *P* < 0.001, adjusted R^2^ = 0.030). Value estimates interacted with time, with larger predicted values associated with greater pupil dilation during late anticipation (t = 4.86, *P* < 0.001, *β* = 0.06) and approach intervals (t = 8.10, *P* < 0.001, *β* = 0.10).

For EEG metrics, the model also provided a significant but modest fit (F [35,155972] = 75.33, *P* < 0.001, adjusted R^2^ = 0.02). Value estimates were associated with oscillatory power across multiple bands and regions. In the alpha band, interactions between learned model value and oscillatory band power were significant over central regions during cue, early, and late intervals (central cue: t = 2.69, *P* = 0.007, *β* = 3.19; central early: t = −2.46, *P* = 0.014, *β* = −2.92; central late: t = −5.32, *P* < 0.001, *β* = −6.33), and over parietal regions during cue and late intervals (parietal cue: t = 5.05, *P* < 0.001, *β* = 6.00; parietal late: t = −4.24, *P* < 0.001, *β* = −5.07). In the beta band, interactions were significant over central regions during early and late intervals (central early: t = −2.33, *P* =0.020, *β* = −2.763; central late: t = −4.13, *P* < 0.001, *β* = −4.89). In the theta band, interactions were significant over frontal regions during cue and early intervals (frontal cue: t = 4.53, *P* < 0.001, *β* = 5.40; frontal early: t = 5.17, *P* < 0.001, *β* = 5.40), and over central scalp regions during early intervals (t = 5.17, *P* < 0.001, *β* = 6.14).

### 3.4. Conditioning results summary

In summary, behavioural indices demonstrated reliable conditioning effects: pain-predictive cues elicited greater pupil dilation and gaze fixations, with anticipatory pupil responses emerging specifically in late time windows. Effects were robust across conditioning and extinction blocks.

Neural conditioning effects were most robustly expressed as anticipatory alpha and beta ERD to pain-predictive cues over central–parietal electrodes. Block effects emerged specifically in frontal–central theta ERS, with stronger ERS during conditioning that diminished during extinction. Effects were time-sensitive, emerging at varying latencies during the trial. Together, these patterns suggest that alpha/beta suppression indexed learned pain predictions, whereas theta synchronisation reflected the overall conditioning context.

Trial-by-trial analyses echoed results from average condition data showing evidence of neural conditioning, with additional observations during earlier CS-US intervals in alpha and theta bands, which were not observed in the standard LMM analyses.

### 3.5. Effects of tonic pain-CS+ congruency

Our central hypothesis was related to whether ongoing tonic pain modulates behavioural or neural responses to pain-predictive cues when both share the same laterality (ie, are congruent). Hence, for these analyses, we looked specifically at the CS+ pain-related cues (ie, not neutral CS− cues) during the extinction block.

For pupil diameter, no significant main effect of congruency or cue × timebin interactions were observed (all *P* > 0.05; Table 1). Thus, congruency effects did not emerge in autonomic responses.

**Figure 5.**
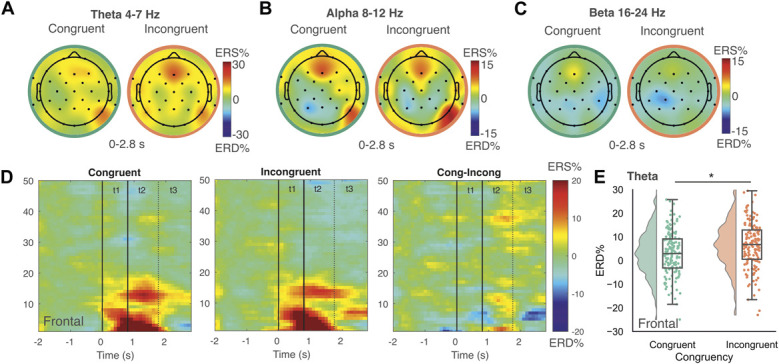
Neural evidence of tonic pain–phasic pain cue congruency during extinction. Topographic maps show ERD/S (event-related desynchronization/synchronization) across the anticipatory period (0-2.8 seconds) in theta (A), alpha (B), and beta (C) frequency bands, comparing trials where tonic pain was applied to a site congruent or incongruent with the cued (CS+) location. Time–frequency plots illustrate relative band power changes for congruent, incongruent, and congruent minus incongruent trials in frontal (D) electrode sites. (E) Significant effects of tonic–phasic pain congruency were found in core analyses in the theta band over frontal sites, with reduced ERS for congruent vs incongruent trials. No congruency effects were observed in alpha or beta bands under this model. Box plots indicate the interquartile range (median = centre line; boxes = 25th–75th percentile; whiskers = values within 1.5 × interquartile range). Negative values indicate ERD; positive values indicate ERS. Asterisks denote significant effects at *P* < 0.05 (family-wise error corrected). CS, conditioned stimulus; ERD, event-related desynchronization; ERS, event-related synchronization.

For EEG activity at the group level (Figs. 5A-E), LMMs showed a significant congruency effect in the *theta band* over frontal electrodes. This was due to a reduced focus of ERS across frontocentral sites for congruent vs incongruent cues, which reached significance following FWE-correction in the frontal regions (t [2134] = 2.62, *P =* 0.008; Fig. 5E). No statistically significant tonic pain congruency effects were observed in alpha or beta bands at the overall group level (*P* > 0.05). Full results are summarized in Table 3.

**Table 3:** Electroencephalography fixed effects for tonic pain—cue congruency and timebin.

Cluster	Predictor	Band														
		Alpha					Beta					Theta				
		*b*	*SE*	*df*	*t* value	*P*	*b*	*SE*	*df*	*t* value	*P*	*b*	*SE*	*df*	*t* value	*P*
Frontal	Intercept	7.47	2.06	80.21	3.63	0.000	2.99	1.25	98.05	2.39	0.019	19.53	2.15	99.45	9.06	0.000
	Congruency	1.75	1.04	861.63	1.68	0.094	0.54	0.66	884.00	0.82	0.413	−3.73	1.16	842.80	−3.23	0.001*
	Timebin	−2.02	0.63	860.91	−3.18	0.002	−1.29	0.41	884.00	−3.17	0.002	−6.45	0.70	837.02	−9.19	0.000
Central	Intercept	0.12	1.83	45.50	0.06	0.949	−0.24	1.00	73.42	−0.24	0.815	14.12	1.35	83.50	10.45	0.000
	Congruency	0.96	0.68	2118.23	1.41	0.158	0.56	0.48	2131.01	1.16	0.245	−0.50	0.68	2088.13	−0.74	0.459
	Timebin	−0.29	0.42	2117.56	−0.68	0.494	−0.90	0.30	2131.01	−3.03	0.002	−4.82	0.42	2086.38	−11.57	0.000
Parietal	Intercept	−8.33	2.14	41.81	−3.90	0.000	−7.55	1.04	53.84	−7.25	0.000	15.13	1.44	72.91	10.54	0.000
	Congruency	0.51	0.75	2658.56	0.68	0.497	−0.34	0.44	2754.01	−0.78	0.438	−0.49	0.68	2685.63	−0.71	0.475
	Timebin	2.34	0.46	2658.48	5.09	0.000	1.80	0.27	2754.00	6.76	0.000	−5.06	0.42	2684.55	−12.05	0.000
Occipital	Intercept	−5.16	2.11	101.85	−2.45	0.016	−5.49	1.30	71.25	−4.24	0.000	16.20	1.97	71.11	8.22	0.000
	Congruency	0.14	1.13	863.79	0.12	0.902	−0.85	0.62	884.00	−1.38	0.169	−0.66	0.94	872.86	−0.71	0.478
	Timebin	1.80	0.69	862.91	2.59	0.010	2.03	0.38	884.00	5.34	0.000	−6.42	0.57	873.41	−11.17	0.000

* Effects that survived FWE correction (*P* < 0.05). Default (treatment) contrast coding was used for measurement timebin (reference: cue) and tonic pain side (reference: incongruent).

FWE, family-wise error.

### 3.6. Computational modelling of trial-by-trial tonic pain-CS+ congruency

Individual linear regression analyses assessed the relationship between EEG/pupil measures and learned model values during extinction blocks with tonic pain. For pupil diameter, the overall fit of the model was modest but statistically significant (F [3,13917] = 9.84, *P* < 0.001, adjusted R^2^ = 0.002); however, no interactions between learned values and congruency were observed. For EEG data, the explanatory power of the model was again small but statistically significant (F [3,61803] = 3.22, *P* = 0.022, adjusted R^2^ = 0.00), showing a steeper positive relationship between *V* and single-trial ERD/S for congruent vs incongruent cues (t = 3.07, *P* = 0.002, *β* = −5.55). When examining this change within frequency bands, there was no significant interaction between learned values, congruency, and band power after FDR correction, although a trend toward a significant effect in the theta band was noted (see Supplementary Materials, http://links.lww.com/PAIN/C449).

### 3.7. Congruency results summary

In summary, tonic–phasic congruency was associated with a reduction in frontal theta ERS. Changes in midfrontal theta ERS were partially captured by trial-by-trial changes in cue valuation.

## 4. Discussion

We investigated how tonic pain influences neural representations of phasic pain predictions using a two-phase integrated EEG-VR revaluation task. Participants were conditioned to predict phasic pain to the left or right arm. EEG data characterised the neural conditioned response, with greater central and parietal alpha and beta ERD associated with pain predictions. In the subsequent extinction phase when tonic pain was applied, we observed reduced midfrontal theta ERS when tonic pain laterality matched (was congruent with) the laterality prediction of phasic pain. Taken together, the data provide neural evidence consistent with internal representations of cue–pain memories which are modulated by tonic pain.

Tonic and phasic pain serve distinct adaptive functions. While phasic pain acts as a rapid alarm and teaching signal for potential harm, tonic pain provides an ongoing homeostatic signal reflecting actual harm and driving protective behaviours.^4,13,68,69^ Tissue damage increases physiological vulnerability in affected regions of the body, prompting protective behaviours to reduce further harm. This adaptive state is reflected in peripheral and central sensitization, where hyperalgesia and allodynia amplify and reshape the perception of new phasic stimuli near the site of damage.^16^ Our findings extend this concept and suggest that the brain adjusts neural predictions of future stimuli in a way that could guide protective responses. These adjustments provide a neural substrate that could in principle allow for preparation for potential harm before it occurs, illustrating the advantage of using learned predictions to optimise behaviour. This capacity to modulate neural representations of predictions according to global physiological priorities is a hallmark of homeostatic processes well-documented for reward systems^24,47^ which were until now proposed but untested in the context of pain.^4,13,60^

Modification of phasic pain predictions by tonic pain implies that the brain forms an internal memory of the cue–pain association that can be modified. This memory must include a topographic representation of the pain location, allowing predicted phasic pain to be internally compared against the sustained pain site. In classical learning theory, modulation of predictions by homeostatic states is a cardinal feature of internal representations or “models,” showing that predictions are not merely cue-driven but hold information specifically relating to the outcome. Revaluation/devaluation paradigms play a central role in animal learning studies, distinguishing “model-based” and “model-free” cognition and decision-making.^18^ Our data provide neural correlates of revaluation effects and suggest that pain learning engages model-based processes in which pain–cue associations are explicitly stored in the brain beyond simple cue-driven or reflexive responses. This adds to evidence for model-based representations for phasic pain, including trace vs delay conditioning^6^ and multistep decision-making.^70^

Neural responses associated with pain predictions can be related to previous studies. During conditioning, pain-predictive cues elicited robust central-parietal alpha and beta suppression after cue offset. Diminished alpha power has been linked to stimulus salience^2,10^ and sustained attention,^34,35^ while beta oscillations have been linked to motor preparation, sensory perception, and selective attention.^19,20,27,42,63^ Consistent with previous aversive conditioning literature,^2^ conditioned responses (CS+ vs CS− cues) were observed and sustained throughout early and late CS-US intervals.

The key modulation in core analyses emerged in response to tonic–phasic pain congruence. Congruent cues—where predicted phasic pain matched the side of tonic pain—evoked diminished frontal theta synchronisation compared with incongruent cues. This effect was absent in simple conditioning contrasts of cue valence (CS+ vs CS–), and overall theta ERS declined during extinction. Thus, the congruency effect cannot be explained as a simple value response. Instead, it suggests that midfrontal theta is sensitive to the computational significance of alignment between ongoing and predicted pain. Previous literature has linked changes in midfrontal theta to cognitive control^8,9^ during conflict and error monitoring,^12,66^ attentional effort,^34,67^ expectancy violation,^7^ and value-based decision-making.^17^ Here, we propose that reduced theta synchronisation for congruent cues may indicate a shift from effortful cognitive control toward more automatic, Pavlovian responding, consistent with theories of control contexts in which diminished theta reflects greater utilisation of Pavlovian control mechanisms when such responses are adaptive.^23^

The observed congruency effect is unlikely to reflect nonspecific changes in arousal or alertness. A global change in arousal would be expected to affect all trials equally within the tonic pain block, whereas theta reductions were specific to within-block differences between congruent and incongruent pain cues. Moreover, our ERD/S analysis used baseline correction within each tonic pain block. As tonic pain was continuous during both baseline and trial intervals, any baseline shift would influence both trial types equally. Finally, physiological indices of arousal showed increases for pain-predictive cues but no main effect of tonic pain laterality, arguing against a pure arousal account.

Trial-by-trial modelling illustrated that changes in pupil diameter and EEG power were predicted by learned cue values, reflecting dynamic updates in pain anticipation. Learned values correlated with pupil dilation during late anticipation and approach intervals. Echoing averaged EEG findings, learned cue values correlated with single-trial EEG in central alpha and beta (early/late intervals) and parietal alpha power (late interval). Modelling analyses also revealed positive correlations between learned values and central and parietal alpha power during cue presentation, and frontal and central theta power during cue and early anticipation intervals. Under tonic pain, EEG oscillations showed a generally stronger negative relationships with learned values for congruent vs incongruent cues. A trend toward this effect was observed in midfrontal theta power, echoing previous literature on Pavlovian influences on action in response inhibition tasks,^23^ although this did not survive correction for multiple comparisons. Further work should seek to clarify the role of theta oscillations in mediating cue valuation processes.

Several caveats should be noted. First, this study did not include a behavioural demonstration that neural evidence of revaluation manifests in enhanced defensive actions, although this is clearly a prediction. Physiological measures showed heightened arousal for pain-predictive cues but not tonic pain laterality. Pupil dilation increased during later anticipation intervals, and fixations were more frequent during the pain–cue approach interval, reflecting heightened arousal for pain-predictive (ie, aversive) cues, consistent with Pavlovian learning.^56,57,59^ Thus, the current results should be interpreted purely as neural correlates of anticipatory processing during associative pain learning, rather than direct evidence of adaptive behavioural change. The observed EEG patterns reflect neural dynamics consistent with preparatory states that may, in future work, be linked to overt defensive responses.

Second, the neural data cannot distinguish encoding of the cue–pain memory from behavioural processes that arise from it. Despite this, the findings imply that some neural encoding of pain memories occurs in the brain. Moreover, devaluation and revaluation paradigms inherently require testing during extinction to avoid contaminating the revalued conditioned response with new learning of directly experienced outcomes (phasic pain). Hence, the Pavlovian memory is expected to decline during the critical test block. This motivates the computational analysis, which captures the trial-by-trial dynamics of how values are expected to diminish throughout the extinction process, but depends on how well the model captures the learning process.

In addition, the sample size was constrained by the technical demands of EEG-VR recording. While sufficient for detecting robust main effects, the study may have been underpowered for higher-order interactions (ie, correlations with subjective pain ratings in Supplementary Materials, http://links.lww.com/PAIN/C449). These analyses should be considered exploratory, and replication with larger samples will be valuable to confirm conditional or subtle effects. Power analyses were not conducted due to the technical complexities of computing power calculations with mixed-effects models, requiring simulation-based approaches (outlined in 36), and this issue is further complicated by the multidimensional nature of neural data. Our approach balanced statistical complexity with stringent methods to reduce type I errors and is in line with previous EEG studies using mixed-methods analyses with comparable sample sizes.^22,28,31,71^ Finally, this study was not pre-registered. Pre-registration can distinguish between established research plans and deviations in the research process due to unexpected outcomes.^38,39^ Future studies could consider pre-registration of core hypotheses and primary outcomes to further enhance transparency and reproducibility.

In summary, the results point toward a novel process by which ongoing tonic pain modulates neural activity associated with predictions of phasic pain in a topographically specific manner. This provides evidence for a specific but previously untested prediction of the broader homeostatic pain hypothesis, illustrating the multifaceted and adaptive nature of pain.

## Conflict of interest statement

The authors have no conflicts of interest to declare.

## Supplementary Material

## Appendix A. Supplemental digital content

Supplemental digital content associated with this article can be found online at http://links.lww.com/PAIN/C449.

## References

[1] Babiloni C Brancucci A Babiloni F Capotosto P Carducci F Cincotti F Arendt-Nielsen L Chen ACN Rossini PM. Anticipatory cortical responses during the expectancy of a predictable painful stimulation. A high-resolution electroencephalography study. Eur J Neurosci 2003;18:1692–700. 14511347 10.1046/j.1460-9568.2003.02851.x

[2] Bacigalupo F Luck SJ. Alpha-band EEG suppression as a neural marker of sustained attentional engagement to conditioned threat stimuli. Social Cogn Affect Neurosci 2022;17:1101–17. 10.1093/scan/nsac029 PMC9766959 35434733

[3] Bates D Mächler M Bolker BM Walker SC. Fitting linear mixed-effects models using lme4. J Stat Softw. 2015;67:1–48.

[4] Bolles RC Fanselow MS. A perceptual-defensive-recuperative model of fear and pain. Behav Brain Sci 1980;3:291–301.

[5] van den Broeke EN van Heck CH van Rijn CM Wilder-Smith O. Neural correlates of heterotopic facilitation induced after high frequency electrical stimulation of nociceptive pathways. Mol Pain 2011;7:28. 21507241 10.1186/1744-8069-7-28 PMC3108312

[6] Carter RMK O'Doherty JP Seymour B Koch C Dolan RJ. Contingency awareness in human aversive conditioning involves the middle frontal gyrus. Neuroimage 2006;29:1007–12. 16246595 10.1016/j.neuroimage.2005.09.011

[7] Cavanagh JF Figueroa CM Cohen MX Frank MJ. Frontal theta reflects uncertainty and unexpectedness during exploration and exploitation. Cereb Cortex 2012;22:2575–86. 22120491 10.1093/cercor/bhr332 PMC4296208

[8] Cavanagh JF Frank MJ. Frontal theta as a mechanism for cognitive control. Trends Cogn Sci 2014;18:414–21. 24835663 10.1016/j.tics.2014.04.012 PMC4112145

[9] Cavanagh JF Shackman AJ. Frontal midline theta reflects anxiety and cognitive control: meta-analytic evidence. J Physiology Paris 2015;109:3–15. 10.1016/j.jphysparis.2014.04.003 PMC4213310 24787485

[10] Chien JH Colloca L Korzeniewska A Cheng JJ Campbell CM Hillis AE Lenz FA. Oscillatory EEG activity induced by conditioning stimuli during fear conditioning reflects salience and valence of these stimuli more than expectancy. Neuroscience 2017;346:81–93. 28077278 10.1016/j.neuroscience.2016.12.047 PMC5426483

[11] Cleland GG Davey GCL. The effects of satiation and reinforcer develuation on signal-centered behavior in the rat. Learn Motiv 1982;13:343–60.

[12] Cohen MX Cavanagh JF. Single-trial regression elucidates the role of prefrontal theta oscillations in response conflict. Front Psychol 2011;2:9539. 10.3389/fpsyg.2011.00030 PMC3111011 21713190

[13] Craig AD. A new view of pain as a homeostatic emotion. Trends Neurosci 2003;26:303–7. 12798599 10.1016/s0166-2236(03)00123-1

[14] Dayan P Berridge KC. Model-based and model-free Pavlovian reward learning: revaluation, revision, and revelation. Cogn Affect Behav Neurosci 2014;14:473–92. 24647659 10.3758/s13415-014-0277-8 PMC4074442

[15] Delorme A Makeig S. EEGLAB: an open source toolbox for analysis of single-trial EEG dynamics including independent component analysis. J Neurosci Methods 2004;134:9–21. 15102499 10.1016/j.jneumeth.2003.10.009

[16] Devor M Wall P. Plasticity in the spinal cord sensory map following peripheral nerve injury in rats. J Neurosci 1981;1:679–84. 7346576 10.1523/JNEUROSCI.01-07-00679.1981 PMC6564200

[17] Diao L Li W Zhang W Ma Q Jin J. Electroencephalographic theta-band oscillatory dynamics represent attentional bias to subjective preferences in value-based decisions. Psychol Res Behav Manag 2021;14:149–58. 33623446 10.2147/PRBM.S292172 PMC7894809

[18] Dickinson A. Contemporary animal learning theory. Cambridge: Cambridge University Press, 1980.

[19] van Ede F De Lange F Jensen O Maris E. Orienting attention to an upcoming tactile event involves a spatially and temporally specific modulation of sensorimotor alpha- and beta-band oscillations. J Neurosci 2011;31:2016–24. 21307240 10.1523/JNEUROSCI.5630-10.2011 PMC6633042

[20] van Ede F Szebényi S Maris E. Attentional modulations of somatosensory alpha, beta and gamma oscillations dissociate between anticipation and stimulus processing. Neuroimage 2014;97:134–41. 24769186 10.1016/j.neuroimage.2014.04.047

[21] Finocchietti S Nielsen M Mørch CD Arendt-Nielsen L Graven-Nielsen T. Pressure-induced muscle pain and tissue biomechanics: a computational and experimental study. Eur J Pain 2011;15:36–44. 20591707 10.1016/j.ejpain.2010.05.010

[22] Frömer R Maier M Abdel Rahman R. Group-level EEG-processing pipeline for flexible single trial-based analyses including linear mixed models. Front Neurosci 2018;12:48. 29472836 10.3389/fnins.2018.00048 PMC5810264

[23] Gershman SJ Guitart-Masip M Cavanagh JF. Neural signatures of arbitration between Pavlovian and instrumental action selection. PLoS Comput Biol 2021;17:e1008553. 33566831 10.1371/journal.pcbi.1008553 PMC7901778

[24] Gottfried JA O'Doherty J Dolan RJ. Encoding predictive reward value in human amygdala and orbitofrontal cortex. Science 2003;301:1104–7. 12934011 10.1126/science.1087919

[25] Graven-Nielsen T Mense S Arendt-Nielsen L. Painful and non-painful pressure sensations from human skeletal muscle. Exp Brain Res 2004;159:273–83. 15480607 10.1007/s00221-004-1937-7

[26] Haegens S Luther L Jensen O. Somatosensory anticipatory alpha activity increases to suppress distracting input. J Cogn Neurosci 2012;24:677–85. 22066587 10.1162/jocn_a_00164

[27] Haegens S Nácher V Hernández A Luna R Jensen O Romo R. Beta oscillations in the monkey sensorimotor network reflect somatosensory decision making. Proc Natl Acad Sci U S A 2011;108:10708–13. 21670296 10.1073/pnas.1107297108 PMC3127887

[28] Hewitt D Besharati S Williams V Leal M McGlone F Stancak A Henderson J Krahé C. Is cultural context the crucial touch? Neurophysiological and self-reported responses to affective touch in women in South Africa and the United Kingdom. Soc Cogn Affect Neurosci 2025:20.nsaf082. 40796184 10.1093/scan/nsaf082 PMC12569768

[29] Hjorth B. An on-line transformation of EEG scalp potentials into orthogonal source derivations. Electroencephalogr Clin Neurophysiol 1975;39:526–30. 52448 10.1016/0013-4694(75)90056-5

[30] Holland PC Rescorla RA. The effect of two ways of devaluing the unconditioned stimulus after first- and second-order appetitive conditioning. J Exp Psychol Anim Behav Process 1975;1:355–63. 1202141 10.1037//0097-7403.1.4.355

[31] Hoy CW Steiner SC Knight RT. Single-trial modeling separates multiple overlapping prediction errors during reward processing in human EEG. Commun Biol 2021;4:910–17. 34302057 10.1038/s42003-021-02426-1 PMC8302587

[32] Jasper HH. The ten-twenty electrode system of the International Federation. Electroencephalogr Clin Neurophysiol. 1958;10:371–5. 10590970

[33] Klein T Magerl W Hopf HC Sandkühler J Treede RD. Perceptual correlates of nociceptive long-term potentiation and long-term depression in humans. J Neurosci 2004;24:964–71. 14749441 10.1523/JNEUROSCI.1222-03.2004 PMC6729815

[34] Klimesch W. EEG alpha and theta oscillations reflect cognitive and memory performance: a review and analysis. Brain Res Rev 1999;29:169–95. 10209231 10.1016/s0165-0173(98)00056-3

[35] Klimesch W Sauseng P Hanslmayr S. EEG alpha oscillations: the inhibition-timing hypothesis. Brain Res Rev 2007;53:63–88. 16887192 10.1016/j.brainresrev.2006.06.003

[36] Kumle L Võ MLH Draschkow D. Estimating power in (generalized) linear mixed models: an open introduction and tutorial in R. Behav Res Methods 2021;53:2528–43. 33954914 10.3758/s13428-021-01546-0 PMC8613146

[37] Kuznetsova A Brockhoff PB Christensen RHB. lmerTest package: tests in linear mixed effects models. J Stat Softw 2017;82:1–26.

[38] Lakens D Mesquida C Rasti S Ditroilo M. The benefits of preregistration and registered reports. Evid Based Toxicol 2024;2:2376046.

[39] Lee H Lamb SE Bagg MK Toomey E Cashin AG Moseley GL. Reproducible and replicable pain research: a critical review. PAIN 2018;159:1683–9. 29697535 10.1097/j.pain.0000000000001254

[40] Lehmann D. Principles of spatial analysis. In: Gevins AS Remond A, editors. Handbook of electroencephalography and clinical neurophysiology: methods of analysis of brain electrical and magnetic signals. Amsterdam: Elsevier, 1987. p. 309–54.

[41] Lelic D Mørch CD Hennings K Andersen OK Drewes AM. Differences in perception and brain activation following stimulation by large versus small area cutaneous surface electrodes. Eur J Pain 2012;16:827–37. 22337577 10.1002/j.1532-2149.2011.00063.x

[42] Lopes da Silva FH Pfurtscheller G. Basic concepts on EEG synchronization and desynchronization. In: Lopes da Silva FH Pfurtscheller G, editors. Handbook of electroencephalography and clinical neurophysiology: event-related desynchronization. Amsterdam: Elsevier Science B.V, 1999. p. 3–12.

[43] Luck SJ. Applied event-related potential data analysis. Davis, CA: LibreTexts; 2022.

[44] Mahajan P Dayan P Seymour B. Homeostasis after injury: how intertwined inference and control underpin post-injury pain and behaviour. PLoS Comput Biol. 2025. doi:10.1101/2025.02.04.636410. In press. PMC12851502 41570047

[45] Mathôt S Fabius J Van Heusden E Van der Stigchel S. Safe and sensible preprocessing and baseline correction of pupil-size data. Behav Res Methods 2018;50:94–106. 29330763 10.3758/s13428-017-1007-2 PMC5809553

[46] May ES Butz M Kahlbrock N Hoogenboom N Brenner M Schnitzler A. Pre- and post-stimulus alpha activity shows differential modulation with spatial attention during the processing of pain. Neuroimage 2012;62:1965–74. 22659486 10.1016/j.neuroimage.2012.05.071

[47] Münch D Ezra-Nevo G Francisco AP Tastekin I Ribeiro C. Nutrient homeostasis—translating internal states to behavior. Curr Opin Neurobiol 2020;60:67–75. 31816522 10.1016/j.conb.2019.10.004

[48] Nyström M Andersson R Niehorster DC Hessels RS Hooge ITC. What is a blink? Classifying and characterizing blinks in eye openness signals. Behav Res Methods 2024;56:3280–99. 38424292 10.3758/s13428-023-02333-9 PMC11133197

[49] Oostenveld R Fries P Maris E Schoffelen J-M. FieldTrip: open source software for advanced analysis of MEG, EEG, and invasive electrophysiological data. Comput Intelligence Neurosci 2011;2011:1–9. 10.1155/2011/156869 PMC3021840 21253357

[50] Perrin F Pernier J Bertrand O Echallier JF. Spherical splines for scalp potential and current density mapping. Electroencephalogr Clin Neurophysiol 1989;72:184–7. 2464490 10.1016/0013-4694(89)90180-6

[51] Pfurtscheller G. Event-related synchronization (ERS): an electrophysiological correlate of cortical areas at rest. Electroencephalogr Clin Neurophysiol 1992;83:62–9. 1376667 10.1016/0013-4694(92)90133-3

[52] Pfurtscheller G. Graphical display and statistical evaluation of event-related desynchronization (ERD). Electroencephalogr Clin Neurophysiol 1977;43:757–60. 72657 10.1016/0013-4694(77)90092-x

[53] Pfurtscheller G Aranibar A. Evaluation of event-related desynchronization (ERD) preceding and following voluntary self-paced movement. Electroencephalogr Clin Neurophysiol 1979;46:138–46. 86421 10.1016/0013-4694(79)90063-4

[54] Pfurtscheller G Aranibar A. Event-related cortical desynchronization detected by power measurements of scalp EEG. Electroencephalogr Clin Neurophysiol 1977;42:817–26. 67933 10.1016/0013-4694(77)90235-8

[55] Pirazzini G Starita F Ricci G Garofalo S di Pellegrino G Magosso E Ursino M. Changes in brain rhythms and connectivity tracking fear acquisition and reversal. Brain Struct Funct 2023;228:1259–81. 37129622 10.1007/s00429-023-02646-7 PMC10250514

[56] Pool ER Pauli WM Cross L O'Doherty JP. Neural substrates of parallel devaluation-sensitive and devaluation-insensitive Pavlovian learning in humans. Nat Commun 2023;14:8057. 38052792 10.1038/s41467-023-43747-5 PMC10697955

[57] Pool ER Pauli WM Kress CS O'Doherty J. Behavioural evidence for parallel outcome-sensitive and outcome-insensitive Pavlovian learning systems in humans. Nat Hum Behav 2019;3:284–96. 30882043 10.1038/s41562-018-0527-9 PMC6416744

[58] Posit Team. RStudio: integrated development environment for R, 2024. Available at: http://www.posit.co/. Accessed March 13, 2024.

[59] Prévost C McNamee D Jessup RK Bossaerts P O'Doherty JP. Evidence for model-based computations in the human amygdala during Pavlovian conditioning. PLoS Comput Biol 2013;9:e1002918. 23436990 10.1371/journal.pcbi.1002918 PMC3578744

[60] Rescorla RA Wagner AR. A theory of Pavlovian conditioning: variations in the effectiveness of reinforcement and non-reinforcement. In: Black AH Prokasy WF, editors. Classical conditioning II: current research theory. Meredith, 1972. p. 64–99. Available at: https://cir.nii.ac.jp/crid/1572543025504096640. Accessed October 11, 2024.

[61] Seropian L Ferschneider M Cholvy F Micheyl C Bidet-Caulet A Moulin A. Comparing methods of analysis in pupillometry: application to the assessment of listening effort in hearing-impaired patients. Heliyon 2022;8:e09631. 35734572 10.1016/j.heliyon.2022.e09631 PMC9207619

[62] Seymour B. Pain: a precision signal for reinforcement learning and control. Neuron 2019;101:1029–41. 30897355 10.1016/j.neuron.2019.01.055

[63] Spitzer B Haegens S. Beyond the status quo: a role for beta oscillations in endogenous content (Re)activation. eNeuro. 2017;4:ENEURO.0170-17.2017. 10.1523/ENEURO.0170-17.2017 PMC5539431 28785729

[64] Starita F Pirazzini G Ricci G Garofalo S Dalbagno D Degni LAE Di Pellegrino G Magosso E Ursino M. Theta and alpha power track the acquisition and reversal of threat predictions and correlate with skin conductance response. Psychophysiology 2023;60:e14247. 36604803 10.1111/psyp.14247

[65] Stern JA Walrath LC Goldstein R. The endogenous eyeblink. Psychophysiology 1984;21:22–33. 6701241 10.1111/j.1469-8986.1984.tb02312.x

[66] Trujillo LT Allen JJB. Theta EEG dynamics of the error-related negativity. Clin Neurophysiol 2007;118:645–68. 17223380 10.1016/j.clinph.2006.11.009

[67] Umemoto A Lin H Holroyd CB. Electrophysiological measures of conflict and reward processing are associated with decisions to engage in physical effort. Psychophysiology 2023;60:e14176. 36097887 10.1111/psyp.14176

[68] Wall PD. On the relation of injury to pain. The John J. Bonica Lecture. PAIN. 1979;6(3):253–64. doi:10.1016/0304-3959(79)90047-2. 460933

[69] Walters ET Williams ACdC. Evolution of mechanisms and behaviour important for pain. Philosophical Trans R Soc B Biol Sci 2019;374:20190275. 10.1098/rstb.2019.0275 PMC6790384 31544614

[70] Wang O Lee SW O'Doherty J Seymour B Yoshida W. Model-based and model-free pain avoidance learning. Brain Neurosci Adv 2018;2:2398212818772964. 30370339 10.1177/2398212818772964 PMC6187988

[71] Zhang B Meng Z Li Q Chen A Bodner GE. EEG-based univariate and multivariate analyses reveal that multiple processes contribute to the production effect in recognition. Cortex 2023;165:57–69. 37267658 10.1016/j.cortex.2023.04.006

[72] Zhang S Mano H Ganesh G Robbins T Seymour B. Dissociable learning processes underlie human pain conditioning. Curr Biol 2016;26:52–8. 26711494 10.1016/j.cub.2015.10.066 PMC4712170

[73] Zhang S Mano H Lee M Yoshida W Kawato M Robbins TW Seymour B. The control of tonic pain by active relief learning. eLife 2018;7:e31949. 29482716 10.7554/eLife.31949 PMC5843408

