# Supplementary material for: Tonic pain modulates neural correlates of associative phasic pain memories
Source: Pain. 2026 Jan 21;167(6):1318–30. doi: 10.1097/j.pain.0000000000003917 (PMC13225244; doi:10.1097/j.pain.0000000000003917)
Supplement: Supplementary file 1 [file jop-167-1318-s001.pdf]

Supplementary Materials

A – Pain Ratings

Table S1. Self-reported ratings and stimulus thresholds prior to the experiment.

		**Detection Intensity**	**Test Intensity**	**Rating Mean (0-10)**
Phasic pain	Left arm	*.08 ± .05 mA*	*1.47 ± 1.2 mA*	***4.85*** *± 1.05*
	Right arm	*.15 ± .13 mA*	*2.12 ± 1.91 mA*	***4.42*** *± 1.27*
Tonic pain	Left arm	*105.00 ± 58.4 bar*	*178.85 ± 76.8 mA*	***3.38*** *± .98*
	Right arm	*93.08 ± 52.82 bar*	*169.62 ± 65.51 mA*	***3.27*** *± .96*

Phasic (electrical) pain

Pain intensity ratings showed a significant main effect of block (F(1,25) =4.31, p = .048) due to lower pain intensity ratings in the second versus the first conditioning block (p = .048). The time-by-block interaction was significant (F(2,50) =6.61, p = .003) due to decreased pain intensity ratings within the second conditioning block (F(2,50) =5.9, p = .005), but not the first (F(2,50) =1.33, p = .273). This effect disappeared after controlling for electrical pain intensity on left and right arms (F=2, 46) = 2.31, p = .111). No significant effects of side, time within block, or other interactions were observed.

Pain unpleasantness ratings significantly decreased within- (F(2,50) =4.27, p = .019) and between-blocks (F(2,25) =19.08, p < .001). This was due to a reduction in unpleasantness ratings as the experiment progressed within blocks (first-last block: p = .036). Reductions between blocks remained significant after accounting for stimulation intensity (F(2,23) = .10.98, p = .003), while within-block changes were no longer significant (F(2,46) = .32, p = .720). No other significant effects were found.

Tonic (pressure) pain

Pain intensity and unpleasantness ratings decreased significantly within-blocks (intensity, F(9,189) = 12.74, p < .001; unpleasantness, F(9,189) = 12.3, p < .001), following a linear trend (intensity, F(1,21) =18.57, p < .001; unpleasantness, F(1,21) =13.07, p = .002). The effect was significant only after the first 2 minutes (time 0 – time 1: intensity, p = .007; unpleasantness, p = .001). Time-by-cuff site effects were significant (intensity, F(9,189) =2.07, p = .034; unpleasantness, F(9,189) =2.16, p = .027), with greater reductions in ratings over time when the cuff was on the right arm (all p < .05) vs. the left arm (all p >.05, except for time 1 vs. 4 for pain unpleasantness). Block-by-time effects showed stronger reductions in intensity for the first versus the second extinction block (intensity, F(9,189) =2.67, p = .006; unpleasantness, F(9,189) =2.53, p = .009). For unpleasantness, the decrease in pain ratings between time 1 and all subsequent timepoints was significant for both blocks (all p < .005). The effect of cuff site was not significant.

Fig. S1.

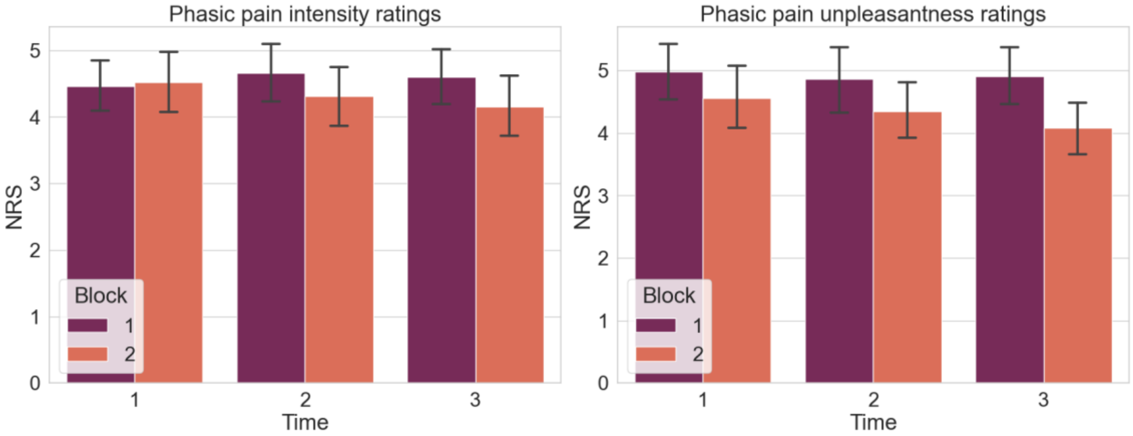

Plot of electrical pain ratings (averaged over left and right sides) during conditioning blocks 1 and 2. Ratings were taken after 24 (Time 1), 48 (2) and 72 trials (3).

Fig. S2.

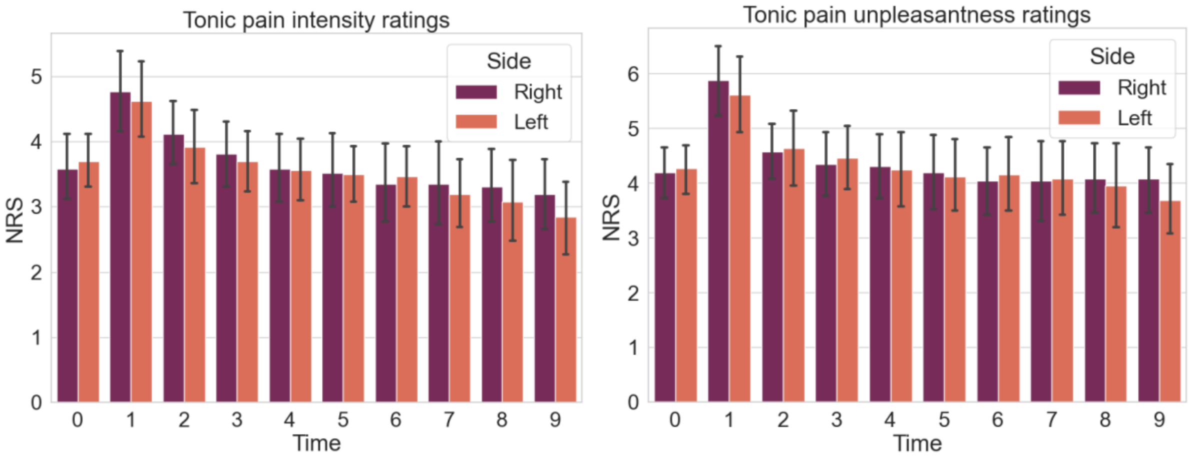

Plot of pressure pain ratings before the experiment (Time 0) and at 2-minute intervals throughout the extinction blocks. Ratings are averaged over each block.

B – Gaze Direction and Hand Position During Pain Anticipation

Gaze direction during each trial was extracted. Instances exceeding 2 SDs for 150 consecutive data points were removed and linearly interpolated. Trials containing >= 25% interpolated data were removed from further analysis (no trials exceeded this threshold). Interpolated data were subject to a median filter (kernel = 5), and the baseline period was removed. The number of fixations on the looming rock was extracted from the last 500 ms of the trial and averaged for each condition. Normalised pupil gaze direction over the entire trial duration showed a point of gaze origin directed slightly towards the right at the start of the trial, followed by a modest leftward shift after cue offset, which returned to the midpoint by the end of the trial (Fig. S3). Results showed no statistically significant effects of cue, block or timebin on gaze direction ($p > .05$). Individual LMMs were used to predict changes in gaze direction with tonic pain, cue congruency and timebin. The main effects of cue congruency and timebin were not significant, nor were there any significant cue × timebin interactions (all $p > .05$).

Hand position from left and right hands was extracted from trial data along x, y and z dimensions and normalised to the starting point for each block. Left- and right-hand positions were averaged to get an overall hand position value for each block, cue type and tonic pain side (for extinction data). Changes in hand position in x, y and z (forward-backwards; up-down; left-right) dimensions were assessed during each condition. Mean displacement during the experiment was x -8.5 ± 20.5 mms, y -6.04 ± 11.96 mms, z 2.56 ± 10.94 mms. A repeated measures ANOVA found no significant difference in mean displacement in any dimension throughout the experiment ($p > .05$).

Fig. S3.

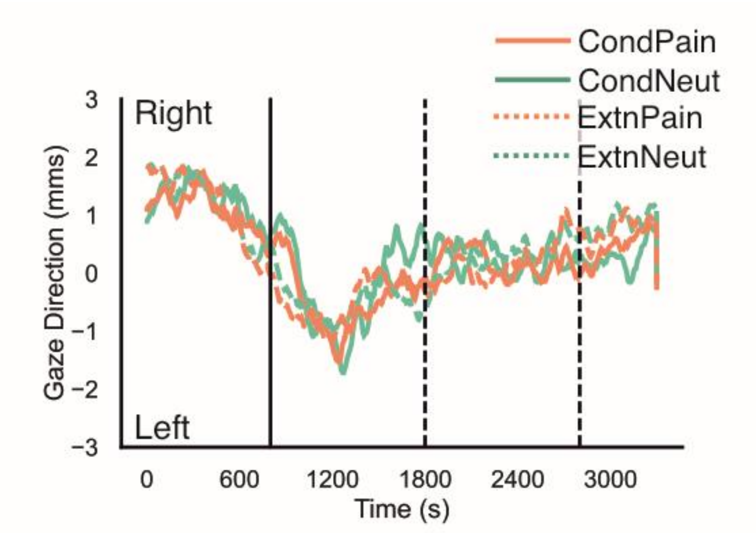

Change in gaze direction throughout the trial, averaged over each condition.

Table S2. Fixed effects for physiological comparisons of interest.

Measure	Predictor	b	SE	df	t value	*p*
**Pupil Diameter** *(Pupil Diameter ~ Cue * Block * Timebin)*	Intercept	-0.04	0.01	367.22	-3.65	0.000
	Cue	-0.03	0.01	1171.86	-2.58	0.010*
	Block	0	0.01	1171.77	-0.21	0.834
	Timebin	0	0	1171.9	0.77	0.441
	Cue × Condition	-0.01	0.01	1171.75	-0.5	0.618
	Cue × Timebin	0.02	0	1171.92	3.68	0.000*
	Block × Timebin	0	0	1171.9	0.06	0.95
	Cue × Block × Timebin	0.01	0	1171.87	1.89	0.059
**Pupil Diameter** *(Pupil Diameter ~ TonicSide + Timebin)*	Intercept	-0.08	0.01	138.66	-7.90	0.000
	Congruency	0.01	0.01	764.67	1.21	0.226
	Timebin	0.02	0	763.96	7.27	0.000*
**Fixations** *(Fixations ~ Cue * Block)*	Intercept	0.07	0.02	45.38	4.13	0.000
	Cue	0.12	0.01	256.47	10.43	0.000*
	Block	0.00	0.01	255.33	-0.14	0.890
	Cue × Block	-0.02	0.02	255.38	-1.20	0.230
**Fixations** *(Fixations ~ TonicPain)*	Intercept	0.19	0.02	27.16	8.06	0.000
	Congruency	-0.01	0.01	154.53	-0.96	0.338
**Gaze Direction** *(Gaze Direction ~ Cue * Block * Timebin)*	Intercept	0.66	2.46	29.46	0.27	0.791
	Cue	-0.18	0.76	1184.97	-0.23	0.819
	Block	0.02	0.62	1185.00	0.03	0.975
	Timebin	-0.15	0.23	1184.97	-0.66	0.511
	Cue × Block	-0.11	0.76	1184.97	-0.14	0.886
	Cue × Timebin	0.06	0.28	1184.97	0.21	0.831
	Block × Timebin	-0.09	0.23	1184.97	-0.42	0.677
	Cue × Block × Timebin	0.07	0.28	1184.97	0.25	0.805
**Gaze Direction** *(Gaze Direction ~ TonicSide + Timebin)*	Intercept	0.24	2.40	26.28	0.10	0.920
	Congruency	0.03	0.36	772.97	0.09	0.932

Asterisks denote effects that survived FWE correction ($p < .05$). Sum contrast coding was used for Block. Default (treatment) contrast coding was used for Cue (reference: neutral), measurement timebin (reference: cue) and tonic pain side (reference: incongruent).

C – Covariate Analyses

Spatial hemisphere bias

The potential spatial hemisphere bias for the tonic pain-cue congruency effect was investigated in EEG data. For assessing the role of electrode hemisphere (contralateral and ipsilateral to the cue) on the pattern of EEG band power, an additional hemisphere factor was included for symmetrical electrodes. As smaller clusters are likely to have smaller degrees of freedom, a broader frontal cluster was used for the assessment of hemisphere lateralisation encompassing F7, F3, F4 and F8, and the occipital region was not included. No lateralised ERD/S effect of cue congruency were observed following FWE-correction (see Table S3).

Combined EEG and Physiological Measures

To examine the additive explanatory value of physiological data to EEG band power changes, pupil diameter and gaze direction were added to LMMs with ERD. Gaze direction did not significantly predict the changes in EEG power spectra during extinction blocks following pain-related cues in any frequency band. Pupil diameter significantly predicted parietal ($F(2,25) = -2.9$, $p < .001$) and occipital beta ($F(2,25) = 19.08$, $p < .001$) and parietal theta ($F(2,25) = 19.08$, $p < .001$) band power. Following FWE-correction, no interactions were found between cue congruency and pupil diameter.

Effects of Tonic Pain-CS+ congruency

Our central hypothesis related to whether ongoing tonic pain modulates behavioural or neural responses to pain-predictive cues when both share the same laterality (i.e., are congruent). Hence, for these analyses, we looked specifically at the CS+ pain-related cues (i.e., not neutral CS- cues) during the extinction block. Additional exploratory analyses looked at the influence of covariates (age, sex, STAI trait scores and mean tonic pain intensity and unpleasantness ratings) on tonic pain-cue congruency.

For pupil diameter, no covariate significantly predicted the change in pupil diameter during anticipation or resulted in a significant congruency effect ($p > .05$). Thus, congruency effects did not emerge in autonomic responses.

For EEG activity at the group level, additional effects of congruency emerged. Accounting for tonic pain ratings resulted in significant main effects of congruency in the ***beta band*** over parietal and occipital sites (Fig. S4), with stronger beta-band ERD for congruent vs. incongruent cues. While this main effect of congruency did not remain significant in pairwise comparisons after correction ($p > .05$), it was qualified by significant congruency × unpleasantness interactions. Participants with the lowest unpleasantness ratings showed attenuated parietal and occipital ERD (/greater ERS) for incongruent vs. congruent cues (parietal, $t(2756) = 3.89$, $p < .001$; occipital, $t(886) = 3.01$, $p = .003$). Conversely, participants reporting the highest tonic pain unpleasantness showed stronger parietal ERD for incongruent cues ($t(2756) = -3.09$, $p = .002$; occipital, $p > .05$). No significant differences were found at mean unpleasantness ratings ($p > .05$).

Fig. S4.

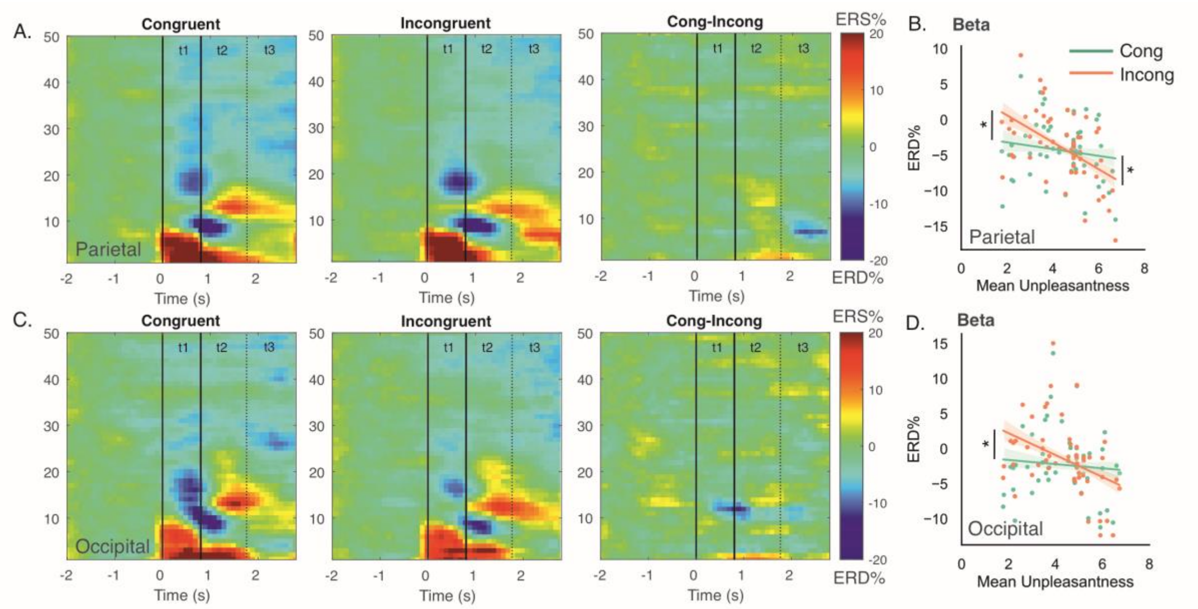

Neural evidence of tonic pain-phasic pain cue congruency during extinction. Exploratory covariate-adjusted analyses accounting for individual differences in tonic pain unpleasantness revealed significant congruency effects in the beta band, specifically in parietal (A-B) and occipital (C-D) regions. These effects were driven by cue × unpleasantness interactions, such that greater unpleasantness was associated with stronger beta ERD for incongruent versus congruent cues. Negative values indicate ERD; positive values indicate ERS. Asterisks denote significant effects at $p < .05$ (FWE-corrected).

Table S3. EEG fixed effects for Tonic pain – cue congruency with covariates.

Band	Predictor	Covariate	Cluster	b	SE	df	t value	p
**Alpha** *(EEG ~ TonicPain * Covariate)*	Congruency	Intensity	Frontal	-8.68	4.10	884.00	-2.12	0.034
	Congruency × Intensity	Intensity	Frontal	2.51	1.08	884.00	2.31	0.021
	Intensity	Intensity	Frontal	-5.68	1.82	54.13	-3.12	0.003*
			Parietal	-5.91	2.09	45.02	-2.83	0.007*
	Congruency	Unpleasantness	Frontal	-13.50	4.13	884.00	-3.27	0.001*
			Central	-6.32	2.43	2132.00	-2.60	0.009*
	Congruency × Unpleasantness	Unpleasantness	Frontal	3.19	0.91	884.00	3.52	0.000*
			Central	1.56	0.54	2132.00	2.91	0.004*
	Unpleasantness	Unpleasantness	Frontal	-5.33	1.54	48.36	-3.46	0.001*
			Central	-3.28	1.28	36.89	-2.57	0.014*
	Congruency × Pupil Diameter	Pupil Diameter	Parietal	21.27	10.33	2787.48	2.06	0.040
	Pupil Diameter	Pupil Diameter	Frontal	-46.18	9.51	927.51	-4.86	0.000*
	Congruency × Unpleasantness × Hemisphere	Unpleasantness + Hemisphere	Frontal	2.58	1.30	1820	1.99	0.047
	Congruency × Hemisphere	Unpleasantness + Hemisphere	Frontal	-11.61	5.90	1820	-1.97	0.049
**Beta** *(EEG ~ TonicPain * Covariate)*	Congruency	Unpleasantness	Parietal	-6.64	1.52	2756.00	-4.36	0.000*
			Occipital	-6.36	2.14	884.00	-2.97	0.003*
		Unpleasantness + Hemisphere	Parietal	-6.76	2.26	2444	-2.99	0.003
			Central	-5.42	2.48	1820	-2.19	0.029
	Congruency × Hemisphere	Pupil Diameter + Hemisphere	Central	-3.37	1.19	1819.85	-2.84	0.005
			Occipital	4.49	1.84	571.95	2.44	0.015
	Congruency × Unpleasantness	Unpleasantness	Parietal	1.42	0.34	2756.00	4.24	0.000*
			Occipital	1.27	0.47	884.00	2.69	0.007*
	Unpleasantness	Unpleasantness	Central	-1.22	0.56	64.98	-2.16	0.034
			Parietal	-1.81	0.59	47.85	-3.06	0.004*
			Occipital	-1.47	0.71	49.57	-2.07	0.044
	Unpleasantness × Hemisphere	Unpleasantness + Hemisphere	Central	1.12	0.54	1820	2.05	0.040
	Congruency × Pupil Diameter	Pupil Diameter	Central	-11.19	5.32	2171.19	-2.10	0.036
	Pupil Diameter	Pupil Diameter	Central	8.33	3.89	2157.72	2.14	0.032
			Parietal	-11.30	3.54	2508.66	-3.20	0.001
			Occipital	-17.17	4.90	919.23	-3.50	0.000*

	Congruency × Unpleasantness	Unpleasantness + Hemisphere	Central	1.55	0.54	1820	2.84	0.005
			Parietal	1.19	0.50	2444	2.40	0.016
	Congruency × Unpleasantness × Hemisphere	Unpleasantness + Hemisphere	Central	-1.92	0.77	1820	-2.49	0.013
	Congruency × Pupil Diameter × Hemisphere	Pupil Diameter + Hemisphere	Central	-24.73	10.40	1820	-2.38	0.018
**Theta** *(EEG ~ TonicPain + Covariate)*	Pupil Diameter	Pupil Diameter	Frontal	-57.21	15.18	733.19	-3.77	0.000*

Note. Asterisks denote effects that survived FWE correction.

D – Computational modelling of trial-by-trial anticipatory changes

Table S4. Learning rates and maximum correlations between learned values and pupil dilation for each participant

ID	Learning rate	Maximum correlation
**2**	0.02	0.15
**3**	0.30	-0.03
**4**	0.31	-0.06
**5**	0.35	0.19
**6**	0.22	0.08
**8**	0.34	0.14
**9**	0.48	-0.03
**10**	0.88	0.13
**11**	0.22	0.07
**12**	0.02	0.02
**13**	0.33	0.18
**14**	0.73	-0.25
**15**	0.01	-0.01
**16**	0.10	0.04
**17**	0.98	-0.02
**19**	0.03	-0.02
**20**	0.99	-0.03
**21**	0.37	0.15
**22**	0.47	0.21
**24**	0.99	0.02
**25**	0.32	0.21
**26**	0.66	0.04
**27**	0.02	0.18
**28**	0.34	0.19
**29**	0.22	0.16
**30**	0.09	0.26

Table S5. Learning rates and maximum correlations between learned values and EEG data for each participant

ID	Learning rate	Maximum correlation
**2**	0.44	-0.06
**3**	0.97	0.10
**4**	0.99	-0.08
**5**	0.99	0.10
**6**	0.04	0.11
**8**	0.99	-0.02
**9**	0.01	0.06
**10**	0.07	0.20
**11**	0.12	0.05
**12**	0.63	0.10
**13**	0.99	-0.03
**14**	0.17	0.41
**15**	0.99	0.02
**16**	0.99	-0.18
**17**	0.99	-0.09
**19**	0.12	0.06
**20**	0.99	-0.04
**21**	0.15	-0.05
**22**	0.99	-0.09
**24**	0.99	-0.11
**25**	0.99	-0.21
**26**	0.43	0.09
**27**	0.13	0.18
**28**	0.99	-0.01
**29**	0.99	-0.16
**30**	0.99	0.03

Table S6. Fixed effects for pupil dilation – Pavlovian learning.

Measure	Predictor	Timebin	b	SE	t value	p
**Pupil Diameter** *(Pupil Diameter ~ V * Timebin)*	Intercept		-0.01	0.003	-2.303	0.021
	Timebin	Early	-0.1	0.005	-19.323	0.000*
		Late	-0.04	0.005	-8.181	0.000*
		Rock	0	0.005	-0.288	0.773
	V		0	0.009	-0.059	0.953
	V × Timebin	Early	0.02	0.013	1.513	0.13
		Late	0.06	0.013	4.862	0.000*
		Rock	0.1	0.013	8.099	0.000*
**Pupil Diameter** *(Pupil Diameter ~ V*Congruency * Timebin)*	Intercept		-0.02	0.008	-2.187	0.029
	Timebin	Early	-0.1	0.011	-9.424	0.000*
		Late	-0.04	0.011	-3.38	0.001*
		Rock	0	0.011	0.302	0.763
	Congruency		0.01	0.011	0.717	0.473
	V		0.01	0.033	0.346	0.729
	V × Congruency		0	0.047	0.091	0.927
	V × Timebin	Early	0.08	0.046	1.821	0.069
		Late	0.03	0.046	0.627	0.53
		Rock	0.07	0.046	1.413	0.158
	V × Timebin × Congruency	Early	0.01	0.015	0.466	0.641
		Late	0	0.015	-0.087	0.931
		Rock	0	0.015	-0.05	0.96

Asterisks denote effects that survived FWE correction ($p < .05$). Reference level for congruency: incongruent.

Table S7. Fixed effects for EEG – Pavlovian learning.

Predictor	Band	Timebin	Location	b	SE	t value	p
**Intercept**				- 1.75	0.120	-14.628	0.000*
**V**				0.14	0.288	0.479	0.632
**Cluster**	Theta	Cue	Frontal	10.04	0.495	20.266	0.000*
			Central	11.05	0.494	22.380	0.000*
		Early	Frontal	3.40	0.496	6.846	0.000*
			Central	1.44	0.493	2.915	0.004*
		Late	Frontal	-4.80	0.496	-9.687	0.000*
			Central	1.04	0.493	2.106	0.035
	Alpha	Cue	Central	1.19	0.494	2.420	0.016*
			Parietal	-4.91	0.495	-9.928	0.000*
		Early	Central	-1.72	0.494	-3.490	0.000*
			Parietal	-7.63	0.495	-15.416	0.000*
		Late	Central	0.88	0.495	1.788	0.074
			Parietal	-1.68	0.498	-3.382	0.001*
	Beta	Cue	Central	0.62	0.492	1.260	0.208
			Parietal	-5.61	0.492	-11.398	0.000*
		Early	Central	-0.35	0.492	-0.713	0.476
			Parietal	-0.99	0.492	-2.007	0.045
		Late	Central	-0.85	0.492	-1.727	0.084
**V × Cluster**	Theta	Cue	Frontal	5.40	1.193	4.526	0.000*
			Central	6.14	1.186	5.174	0.000*
		Early	Frontal	5.23	1.194	4.384	0.000*
			Central	2.53	1.186	2.130	0.033
		Late	Frontal	1.07	1.190	0.895	0.371
			Central	-0.74	1.187	-0.625	0.532
	Alpha	Cue	Central	3.19	1.188	2.687	0.007*
			Parietal	6.00	1.190	5.045	0.000*
		Early	Central	-2.92	1.187	-2.458	0.014*
			Parietal	-1.73	1.189	-1.457	0.145
		Late	Central	-6.33	1.188	-5.324	0.000*
			Parietal	-5.07	1.195	-4.239	0.000*
	Beta	Cue	Central	0.27	1.184	0.225	0.822
			Parietal	0.90	1.184	0.763	0.445
		Early	Central	-2.76	1.184	-2.333	0.020*
			Parietal	-1.73	1.184	-1.458	0.145
		Late	Central	-4.89	1.185	-4.127	0.000*

Formula: EEG ~ V * Cluster. Asterisks denote effects that survived FWE correction ($p < .05$). Reference level for congruency: incongruent.

Table S8. Fixed effects for EEG – Pavlovian learning, Congruency Effects.

Predictor	Band	Timebin	Location	b	SE	t value	p
**Intercept**				-2.48	0.253	-9.824	0.000*
**Congruency**				-0.47	0.357	-1.328	0.184
**V**				-2.26	1.251	-1.806	0.071
**V × Congruency**				5.58	1.795	3.111	0.002*
**Cluster**	Theta	Cue	Frontal	10.74	1.047	10.262	0.000*
			Central	10.74	1.043	10.294	0.000*
		Early	Frontal	2.66	1.049	2.537	0.011
			Central	0.85	1.04	0.816	0.414
		Late	Frontal	-5.89	1.049	-5.617	0.000*
			Central	0.28	1.042	0.269	0.788
	Alpha	Cue	Central	1.39	1.041	1.338	0.181
			Parietal	-3.76	1.043	-3.605	0.000*
		Early	Central	-2.07	1.039	-1.99	0.047
			Parietal	-6.69	1.044	-6.41	0.000*
		Late	Central	0.93	1.043	0.891	0.373
			Parietal	-0.85	1.052	-0.808	0.419
	Beta	Cue	Central	-0.05	1.04	-0.052	0.958
			Parietal	-5.65	1.038	-5.446	0.000*
		Early	Central	-0.7	1.037	-0.677	0.499
			Parietal	-1.15	1.037	-1.112	0.266
		Late	Central	-1.04	1.037	-1.002	0.317
**V × Cluster**	Theta	Cue	Frontal	0.98	5.165	0.19	0.849
			Central	5.44	5.159	1.055	0.291
		Early	Frontal	19.6	5.176	3.787	0.000*
			Central	7.35	5.15	1.427	0.154
		Late	Frontal	16.27	5.169	3.147	0.002*
			Central	3.41	5.162	0.66	0.509
	Alpha	Cue	Central	-2.34	5.157	-0.454	0.65
			Parietal	-3.47	5.16	-0.673	0.501
		Early	Central	-6.15	5.149	-1.195	0.232
			Parietal	-9.19	5.153	-1.784	0.074
		Late	Central	-18.3	5.18	-3.535	0.000*
			Parietal	-16.15	5.182	-3.117	0.002*
	Beta	Cue	Central	3.49	5.153	0.678	0.498
			Parietal	10.72	5.149	2.083	0.037
		Early	Central	-7.06	5.151	-1.371	0.171
			Parietal	1.01	5.151	0.197	0.844
		Late	Central	-2.5	5.149	-0.485	0.628
**Cluster × Congruency**	Theta	Cue	Frontal	-4.57	1.476	-3.095	0.002*
			Central	-0.29	1.47	-0.2	0.841

		Early	Frontal	-1.76	1.479	-1.192	0.233
			Central	0.43	1.468	0.292	0.77
		Late	Frontal	-0.81	1.479	-0.547	0.585
			Central	1.83	1.47	1.244	0.214
	Alpha	Cue	Central	0.91	1.469	0.616	0.538
			Parietal	-1.66	1.473	-1.128	0.259
		Early	Central	0.94	1.468	0.639	0.523
			Parietal	-0.07	1.474	-0.046	0.963
		Late	Central	0.8	1.472	0.547	0.585
			Parietal	0.09	1.483	0.061	0.951
	Beta	Cue	Central	1.58	1.466	1.077	0.281
			Parietal	0.43	1.465	0.293	0.769
		Early	Central	1.61	1.464	1.102	0.27
			Parietal	0.75	1.463	0.513	0.608
		Late	Central	0.86	1.465	0.588	0.557
**V × Cluster × Congruency**	Theta	Cue	Frontal	16.7	7.405	2.255	0.024
			Central	-2.36	7.394	-0.319	0.75
		Early	Frontal	5.67	7.436	0.762	0.446
			Central	-3.4	7.396	-0.459	0.646
		Late	Frontal	-1.49	7.444	-0.2	0.841
			Central	-12.28	7.402	-1.659	0.097
	Alpha	Cue	Central	-0.78	7.398	-0.105	0.916
			Central	8.07	7.406	1.089	0.276
		Early	Parietal	-0.22	7.392	-0.03	0.976
			Central	0.24	7.397	0.032	0.974
		Late	Parietal	4.45	7.413	0.6	0.548
			Central	-3.41	7.425	-0.459	0.646
	Beta	Cue	Central	-2.8	7.395	-0.379	0.705
			Parietal	-1.78	7.388	-0.24	0.81
		Early	Central	3.15	7.4	0.426	0.67
			Parietal	-1.39	7.388	-0.188	0.851
		Late	Central	-4.3	7.386	-0.583	0.56

Formula: EEG ~ V * Congruency * Cluster. Asterisks denote effects that survived FWE correction ($p < .05$). Reference level for congruency: incongruent.
